# The mTOR Inhibitor Rapamycin Prevents General Anesthesia-Induced Changes in Synaptic Transmission and Mitochondrial Respiration in Late Postnatal Mice

**DOI:** 10.3389/fncel.2020.00004

**Published:** 2020-01-28

**Authors:** Xianshu Ju, Min Jeong Ryu, Jianchen Cui, Yulim Lee, Sangil Park, Boohwi Hong, Sungho Yoo, Won Hyung Lee, Yong Sup Shin, Seok-Hwa Yoon, Gi Ryang Kweon, Yoon Hee Kim, Youngkwon Ko, Jun Young Heo, Woosuk Chung

**Affiliations:** ^1^Department of Biochemistry, Chungnam National University School of Medicine, Daejeon, South Korea; ^2^Department of Medical Science, Chungnam National University School of Medicine, Daejeon, South Korea; ^3^Infection Control Convergence Research Center, Chungnam National University School of Medicine, Daejeon, South Korea; ^4^Department of Anesthesia and Pain Medicine, Chungnam National University Hospital, Daejeon, South Korea; ^5^Department of Anesthesia and Pain Medicine, Chungnam National University School of Medicine, Daejeon, South Korea

**Keywords:** general anesthesia, mTOR, neurodevelopment, neurotoxicity, synaptic transmission

## Abstract

Preclinical animal studies have continuously reported the possibility of long-lasting neurotoxic effects after general anesthesia in young animals. Such studies also show that the neurological changes induced by anesthesia in young animals differ by their neurodevelopmental stage. Exposure to anesthetic agents increase dendritic spines and induce sex-dependent changes of excitatory/inhibitory synaptic transmission in late postnatal mice, a critical synaptogenic period. However, the mechanisms underlying these changes remain unclear. Abnormal activation of the mammalian target of rapamycin (mTOR) signaling pathway, an important regulator of neurodevelopment, has also been shown to induce similar changes during neurodevelopment. Interestingly, previous studies show that exposure to general anesthetics during neurodevelopment can activate the mTOR signaling pathway. This study, therefore, evaluated the role of mTOR signaling after exposing postnatal day (PND) 16/17 mice to sevoflurane, a widely used inhalation agent in pediatric patients. We first confirmed that a 2-h exposure of 2.5% sevoflurane could induce widespread mTOR phosphorylation in both male and female mice. Pretreatment with the mTOR inhibitor rapamycin not only prevented anesthesia-induced mTOR phosphorylation, but also the increase in mitochondrial respiration and male-dependent enhancement of excitatory synaptic transmission. However, the changes in inhibitory synaptic transmission that appear after anesthesia in female mice were not affected by rapamycin pretreatment. Our results suggest that mTOR inhibitors may act as potential therapeutic agents for anesthesia-induced changes in the developing brain.

## Introduction

Preclinical animal studies continuously report possible neurotoxic effects from anesthesia in young rodents, sheep, and non-human primates (Olutoye et al., 2016; Jevtovic-Todorovic, 2018). The United States Food and Drug Administration (U.S. FDA) has therefore published warnings regarding the repeated or prolonged use of anesthesia in children under age 3 years. Fortunately, recent clinical studies strongly suggest that a single, short exposure to anesthetic does not affect neurodevelopment (O’Leary and Warner, 2017; Warner et al., 2018; McCann et al., 2019). However, there are still concerns regarding multiple anesthetic exposures (Warner et al., 2018; Zaccariello et al., 2019).

Previous studies also show that anesthesia-induced neurotoxicity depends on their neurodevelopmental stage. While anesthesia induces neuronal cell death in neonatal mice, the same anesthetics induces excitatory/inhibitory imbalance in late postnatal mice (Briner et al., 2010; Chung et al., 2017a; Ju et al., 2019). Importantly, excitatory/inhibitory imbalance has been linked to diverse neurodevelopmental disorders (Meredith, 2015; Lee et al., 2017). Because most procedures requiring anesthesia in humans are performed during the postnatal period, these anesthesia-induced changes in late postnatal mice may be of great importance, as the neurodevelopment of mice during this stage may be equivalent to the neurodevelopment of human infants (Workman et al., 2013). However, the mechanisms underlying anesthesia-induced changes in late postnatal mice are still not completely understood.

The mammalian target of rapamycin (mTOR), a serine/threonine kinase, controls intra-cellular functions including protein synthesis, energy metabolism, cell survival, autophagy and mitochondria biogenesis in peripheral tissues. In the nervous system, mTOR pathway regulates axonal sprouting, axonal regeneration and myelination, ion channel and receptor expression, and dendritic spine growth (Bockaert and Marin, 2015; Huber et al., 2015). Previous studies also show that activation of mTOR enhances synaptic activity by promoting AMPA receptor synthesis and expression at the cell surface (Wang et al., 2006; Ran et al., 2013). In addition, mTOR regulates dendritic spine development and formation (Tavazoie et al., 2005; Lee et al., 2011), and excitatory synaptic transmission (Tang et al., 2002; Cammalleri et al., 2003). As exposure to anesthetics increase synaptic proteins, dendritic spinogenesis, and induce excitatory/inhibitory imbalance (Briner et al., 2010; Chung et al., 2017a; Ju et al., 2019), it is highly possible that mTOR signaling is involved with these anesthesia-induced changes. Indeed, previous studies show that anesthetics increase mTOR signaling in various ages (Li et al., 2010, 2017; Zhang et al., 2014; Kang et al., 2017). For example, injection of ketamine in adult mice was found to induce mTOR activation, accompanied by increased spinogenesis and excitatory synaptic transmission (Li et al., 2010). Isoflurane induction of anesthesia in postnatal day (PND) 15 mice was found to induce long-lasting mTOR pathway activation in the dentate gyrus, leading to changes in dendritic arbors, dendritic spines numbers, and impaired learning and memory (Kang et al., 2017). These changes were prevented by the mTOR pathway inhibitor rapamycin. However, the exact role of mTOR after general anesthesia in late postnatal mice has not been sufficiently evaluated.

To evaluate the role of mTOR signaling following anesthesia in late postnatal mice, PND 16/17 mice were exposed to 2.5% sevoflurane (the most widely used inhalation agent in pediatric patients) for 2 h. Administration of sevoflurane to PND 16/17 mice has been shown to increase dendritic spine formation, to alter mitochondrial function, and to induce sex-dependent changes in excitatory/inhibitory synaptic transmission (Chung et al., 2017a; Ju et al., 2019). Based on previous findings, this study hypothesized that sevoflurane-induced changes in late postnatal mice could be prevented by inhibiting the mTOR pathway with rapamycin.

## Materials and Methods

### Animals

All experiments were approved by the relevant Committees of Chungnam National University, Daejeon, South Korea (CNU-01135). C57BL/6J mice were maintained in a specific pathogen-free (SPF) room maintained at 22°C, with a 12 h light/dark cycle, and fed *ad libitum*. Animals received anesthesia during the light cycle. This research adheres to the ARRIVE (Animal Research: Reporting *in vivo* Experiments) guidelines.

### Anesthesia

PND 16/17 mice were randomly divided into three groups: control, sevoflurane, and sevoflurane plus rapamycin groups. Mice in the sevoflurane and sevoflurane plus rapamycin groups were placed in a 1-l plastic chamber and exposed to a constant flow of fresh gas [fraction of inspired oxygen (FiO_2_) 0.4, 4 L/min] containing 2.5% sevoflurane for 2 h. Full recovery was confirmed 30 min after discontinuing sevoflurane. Control mice were treated identically but without sevoflurane. The anesthesia chamber was placed in a 36°C water bath to maintain a constant temperature. Carbon dioxide and sevoflurane were monitored using an S/5 compact anesthetic monitor and a mCAiO gas analyzer module (Datex-Ohmeda, Helsinki, Finland).

### Rapamycin Treatment

Rapamycin (LC Laboratories, Woburn, MA, USA) was reconstituted in ethanol at a concentration 10 μg/μl and then diluted in 5% Tween-80 (Sigma–Aldrich, St. Louis, MO, USA) and 5% PEG-400 (Sigma–Aldrich, St. Louis, MO, USA), as described (Chen et al., 2009). Mice in the sevoflurane plus rapamycin group were each administered three intraperitoneal injections of rapamycin (5 mg/kg) at 24 h intervals prior to sevoflurane exposure, whereas mice in the control and sevoflurane groups were injected with an identical volume of vehicle.

### Western blotting

Whole-brain samples were obtained from the mice 24 h after sevoflurane exposure. Mice were exposed to carbon dioxide before brain extraction, and each whole brain was homogenized with a tissue grinder in RIPA lysis buffer [ELPIS-BIOTECH, Daejeon, South Korea, 100 mM Tris–hydrochloride (pH 8.5), 200 mM NaCl, 5 mM EDTA, and 0.2% sodium dodecyl sulfate], containing phosphatase and protease inhibitor cocktails (Sigma–Aldrich). After centrifuging the homogenized samples at 12,000× *g* for 15 min at 4°C, the supernatants were decanted and their protein concentrations were measured using the Bradford assay (Bio-Rad, Hercules, CA, USA). Samples (20 μg) were electrophoresed on SDS PAGE gels, and transferred to nitrocellulose membranes (pore size, 0.2 μm; Amersham Protran^®^, GE Healthcare, Buckinghamshire, UK) at 200 mA for 2 h. The membranes were blocked for 1 h with Tris-buffered saline-Tween 20 [10 mM Tris–hydrochloride (pH 7.6), 150 mM NaCl, and 0.1% Tween 20], containing 3% bovine serum albumin (BSA), followed by incubation with primary antibodies and the appropriate secondary antibodies coupled to horseradish peroxidase. Specific antibody-labeled proteins were detected using the enhanced chemiluminescence system (WEST-ZOL plus; iNtRON BioTechnology, Seongnam, South Korea). Primary antibodies included antibodies to phospho-mTOR(S2448), mTOR (Cell Signaling Technology, Danvers, MA, USA), postsynaptic density 90 (PSD95; Neuromab, Davis, CA, USA), GAD65 (Abcam, Cambridge, UK), NDUFB8 (a mitochondrial complex I subunit; Santa Cruz Biotechnology, Santa Cruz, TX, USA), COX4 (a mitochondrial complex IV subunit; Novus Biologicals, Centennial, CO, USA) and actin (Santa Cruz Biotechnology, Santa Cruz, TX, USA). Antibodies against GluA1 (1193) and GluA2 (1195) have been described previously (Kim et al., 2009).

### Oxygen Consumption Rate

Mitochondria were isolated from brain tissues 24 h after sevoflurane exposure, as previously described (Chung et al., 2017a). Each brain was homogenized in a mitochondrial isolation buffer [70 mM sucrose, 210 mM mannitol, 5 mM HEPES, 1 mM EGTA, and 0.5% (w/v) fatty acid–free BSA (pH 7.2)] with a Teflon-glass homogenizer (Thomas Fisher Scientific, Swedesboro, NJ, USA). After centrifugation at 600× *g* for 10 min at 4°C and at 17,000× *g* for 10 min at 4°C, the mitochondrial fraction was resuspended in a mitochondrial isolation buffer. Protein concentration was measured by the Bradford assay (Bio-Rad), and 20 μg aliquots of protein were diluted with 50 μl mitochondrial assay solution [70 mM sucrose, 220 mM mannitol, 10 mM KH_2_PO_4_, 5 mM MgCl_2_, 2 mM HEPES, 1 mM EGTA, 0.2% (w/v) fatty acid–free BSA, 10 mM succinate, and 2 μM rotenone (pH 7.2)] and seeded in an XF-24 plate (Seahorse Bioscience, North Billerica, MA, USA). The plates were centrifuged at 2,000× g for 20 min at 4°C using a swinging bucket microplate adaptor (Eppendorf, Hamburg, Germany); 450 μl mitochondrial assay buffer was added to each plate, and the plates were maintained at 37°C for 8–10 min. Each plate was transferred to a Seahorse XF-24 extracellular flux analyzer (Seahorse Bioscience) and the oxygen consumption rate (OCR) was measured at five stages: stage I (basal level); stage II, following the addition of adenosine diphosphate (ADP); stage III, following the addition of oligomycin, a mitochondrial oxidative phosphorylation (OXPHOS) complex 5 inhibitor; stage IV, following the addition of carbonyl cyanide m-chlorophenyl hydrazine (CCCP), a mitochondrial OXPHOS complex 4 inhibitor; and stage V, following the addition of antimycin A, a mitochondrial OXPHOS complex 3 inhibitor. OCR was automatically calculated and recorded using Seahorse XF-24 software (Seahorse Bioscience).

### Electrophysiology

Whole-cell voltage-clamp recordings of pyramidal neurons in the CA1 region of the hippocampus were obtained as described (Chung et al., 2015a). Twenty-four hours after exposure to sevoflurane or fresh gas, sagittal slices of the hippocampus (300 μm) were prepared in ice-cold dissection buffer (212 mM sucrose, 25 mM NaHCO_3_, 5 mM KCl, 1.25 mM NaH_2_PO_4_, 10 mM d-glucose, 2 mM sodium pyruvate, 1.2 mM sodium ascorbate, 3.5 mM MgCl_2_, and 0.5 mM CaCl_2_) aerated with 95% O_2_/5% CO_2_, using a VT1200S vibratome (Leica, Arrau, Switzerland). Slices were transferred immediately to a 32°C chamber containing artificial cerebrospinal fluid (aCSF: 125 mM NaCl, 25 mM NaHCO_3_, 2.5 mM KCl, 1.25 mM NaH_2_PO_4_, 10 mM d-glucose, 1.3 mM MgCl_2_, and 2.5 mM CaCl_2_, continuously aerated with 95% O_2_/5% CO_2_) and incubated for 30 min. Glass capillaries were filled with two kinds of internal solutions. For miniature excitatory postsynaptic current (mEPSC) recordings, the glass capillaries were filled with an internal solution containing 117 mM CsMeSO_4_, 10 mM tetraethylammonium chloride, 8 mM NaCl, 10 mM HEPES, 5 mM QX-314-Cl, 4 mM Mg-adenosine triphosphate (ATP), 0.3 mM Na-guanosine triphosphate, and 10 mM EGTA; for miniature inhibitory postsynaptic current (mIPSC) recordings, the glass capillaries were filled with an internal solution containing 115 mM CsCl, 10 mM tetraethylammonium chloride, 8 mM NaCl, 10 mM HEPES, 5 mM QX-314-Cl, 4 mM Mg-ATP, 0.3 mM Na-guanosine triphosphate, and 10 mM EGTA. Whole-cell recordings were performed under visual control (BX50WI; Olympus, Tokyo, Japan) with a multi clamp 700A amplifier (Molecular Devices, San Jose, CA, USA). Data were acquired with Clampex 9.2 (Molecular Devices, San Jose, CA, USA) and analyzed using Clampfit 9 software (Molecular Devices, San Jose, CA, USA).

### Statistical Analysis

The sample size was determined based on previous experience or as previously described (Chung et al., 2015b, 2017b). All statistical analyses were performed using R statistical software (3.1.2: R Core Team, Austria). All continuous variables were tested to determine whether they met conditions of normality and homogeneity of variance. One-way ANOVA with *post hoc* Tukey HSD test was performed when both conditions were met, Welch’s ANOVA with *post hoc* Tukey HSD test was performed when homogeneity of variance was unmet, and the Kruskal–Wallis test with *post hoc* Dunn’s test was performed if normality was unmet. *P* < 0.05 was considered statistically significant. Statistical results are presented as Supplementary Statistics.

## Results

### Sevoflurane Exposure in PND 16/17 Mice Induces Widespread Activation of the mTOR Signaling Pathway

**Figure 1:**
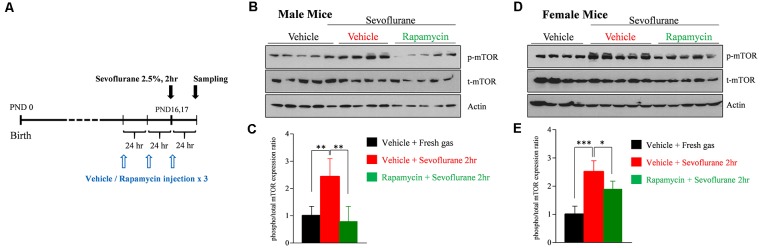
Rapamycin prevents sevoflurane-induced activation of mammalian target of rapamycin (mTOR) signaling in male and female postnatal day (PND) 16/17 mice. **(A)** Time schedule for experiments. PND 14/15 mice were intraperitoneally injected with vehicle or rapamycin once daily for 3 days. On day 3 (PND 16/17), the mice were exposed to 2.5% sevoflurane anesthesia for 2 h. On day 4, mice were sacrificed and their whole brains were extracted. **(B–E)** Western blot of whole-brain samples obtained 24 h after sevoflurane exposure. **(B,D)** Western blotting with antibodies specific for phosphorylated and total mTOR and actin (loading control) in male and female mice. **(C,E)** Quantification of mean ± SD protein band intensity in panels (**B,D**; *n* = 4 or 5 per group; *p < 0.05, **p < 0.01, ***p < 0.001).

To assess that sevoflurane induces widespread activation of the mTOR signaling pathway, the level of mTOR phosphorylation was measured by western blotting in whole brain samples obtained 24 h after sevoflurane exposure. We also evaluated whether three daily injections of rapamycin prior to sevoflurane exposure could prevent mTOR phosphorylation (Figure 1A). Because we had found that sevoflurane changes were sex-dependent, mTOR phosphorylation was separately measured in male and female mice. Sevoflurane exposure enhanced phosphorylation of mTOR in both male and female mice, and such phosphorylation was blocked in both sexes by rapamycin injection (Figures 1B–E).

### Rapamycin Treatment Prevents a Sevoflurane-Induced Increase of Mitochondrial Function in PND 16/17 Mice

**Figure 2:**
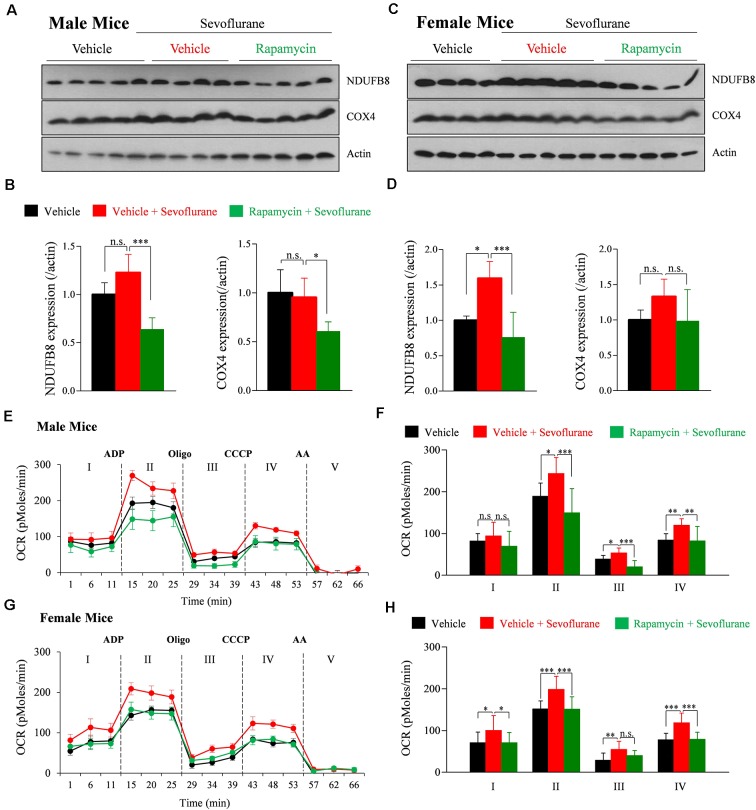
Rapamycin prevents sevoflurane-induced increases in mitochondrial function and oxidative phosphorylation (OXPHOS) complexes in PND 16/17 mice. **(A–D)** Whole-brain samples obtained 24 h after sevoflurane exposure. **(A,C)** Western blotting with antibodies specific for the OXPHOS complex subunits NDUFB8 (OXPHOS complex I) and COX4 (OXPHOS complex IV) and actin (loading control) in male and female mice. **(B,D)** Quantification of mean ± SD protein band intensity in panels (**A,C**; *n* = 4 or 5 per group; n.s., not significant, *p < 0.05, ***p < 0.01). **(E–H)** Oxygen consumption rate (OCR) of mitochondria isolated from the whole brain 24 h after sevoflurane exposure. The substrate was used by adding succinate to the mitochondrial assay buffer. Adenosine diphosphate (ADP) was used to stimulate adenosine triphosphate (ATP) turnover and ATP generation was measured with oligomycin (Oligo). Maximal OCR was assessed using carbonyl cyanide m-chlorophenyl hydrazone (CCCP) and non-mitochondrial OCR was measured using antimycin A (AA; *n* = 4 or 5 per group; n.s., not significant, *p < 0.05, **p < 0.01, ***p < 0.001). Values are presented as mean ± SD.

We previously reported that mitochondrial respiration continuously increases for up to 9 h after sevoflurane exposure in PND 16/17 mice (Chung et al., 2017a). Mitochondrial respiration, which is conducted by assembled mitochondrial and nuclear-originated proteins, produces ATP by consuming oxygen. To determine the association between mTOR signaling and sevoflurane-induced changes in mitochondrial respiration, the amounts of the OXPHOS complex subunit proteins NADH: Ubiquinone Oxidoreductase Subunit B8 (NDUFB8; subunit of OXPHOS complex I) and cytochrome c oxidase subunit 4 (COX4; subunit of OXPHOS complex IV) were measured 24 h after sevoflurane exposure. Sevoflurane increased the level of NDUFB8, but not of COX4, only in female mice. The increase was inhibited by preinjection with rapamycin (Figures 2A–D). To assess mitochondrial respiration 24 h after sevoflurane exposure, we also measured the OCR in mitochondria isolated from whole brains (Figures 2E–H). Sevoflurane exposure increased basal OCR (stage I) only in female mice, but increased ADP-induced OCR (stage II), oligomycin induced ATP production (stage III), and maximal OCR (stage IV) in both male and female mice. These changes were prevented by rapamycin pretreatment in both male and female mice (Figures 2F,H). Taken together, these results showed that sevoflurane-induced changes in mitochondrial function in an mTOR dependent manner.

### Rapamycin Treatment Prevents a Sevoflurane-Induced Increase of AMPA Receptor Subunit GluA2 in PND 16/17 Male Mice

**Figure 3:**
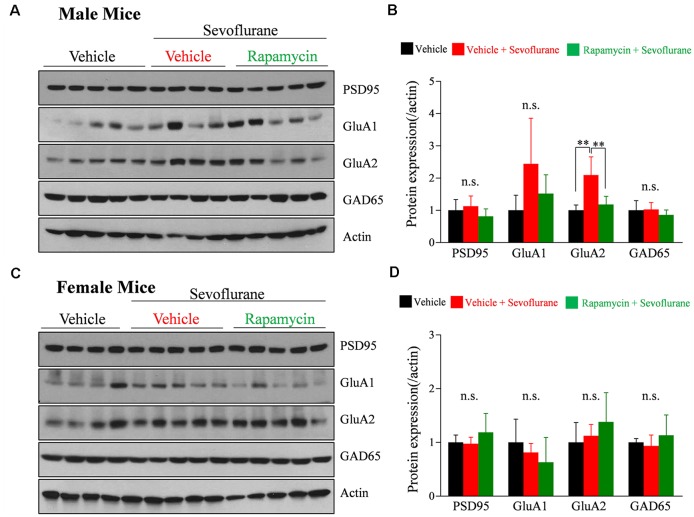
Rapamycin prevents the sevoflurane-induced increase in the expression of the α-amino-3-hydroxy-5-methyl-4-isoxazolepropionate (AMPA) receptor subunit GluA2 in PND 16/17 male mice. **(A–D)** Western blotting of whole-brain samples obtained 24 h after sevoflurane exposure for expression of excitatory {postsynaptic density 95 [PSD95], GluA1, GluA2} and inhibitory (GAD65) synaptic proteins. Actin was used as the loading control. **(B,D)** Mean ± SD protein band intensity in panels (**A,C**; *n* = 4 or 5 per group; n.s., not significant, **p < 0.01).

We previously reported that a single exposure of PND 16/17 mice to sevoflurane affects the level of expression of synaptic molecules 6 h later (Chung et al., 2017a; Ju et al., 2019). To confirm longer-lasting changes in expression and to determine the role of mTOR signaling, western blotting was performed 24 h after sevoflurane exposure. The expression of GluA2 was significantly increased only in male mice, while there were no significant changes in female mice (Figure 3). The increase in GluA2 expression was inhibited by rapamycin pretreatment, suggesting that the mTOR pathway is also associated with changes in protein expression after exposure to sevoflurane in male mice.

### Rapamycin Treatment Prevents Sevoflurane-Induced Changes of Excitatory Synaptic Transmission in Male Mice But Does Not Prevent Changes of Inhibitory Synaptic Transmission in Female Mice

**Figure 4:**
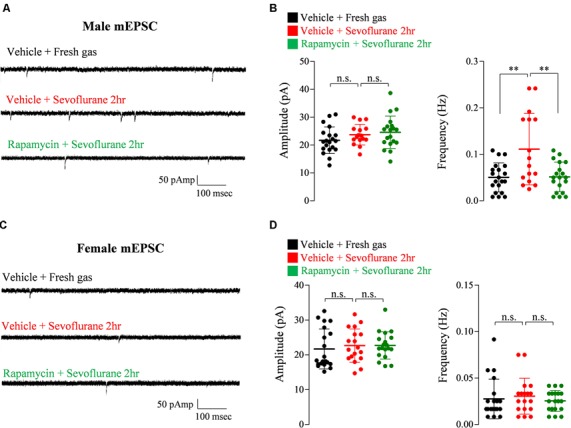
Rapamycin prevents the increase in excitatory synaptic transmission in PND 16/17 male mice 24 h after sevoflurane exposure. **(A,B)** Frequency and amplitude of miniature excitatory postsynaptic currents (mEPSCs) in male mice 24 h after sevoflurane exposure with/without rapamycin pretreatment (control: 20 cells from three mice; sevoflurane: 16 cells from three mice; rapamycin: 19 cells from three mice; n.s., not significant, **p < 0.01). **(C,D)** Frequency and amplitude of mEPSC in female mice 24 h after sevoflurane exposure with/without rapamycin pretreatment (control: 20 cells from three mice; sevoflurane: 18 cells from three mice; rapamycin: 19 cells from three mice; n.s., not significant). Values are presented as mean ± SD.

**Figure 5:**
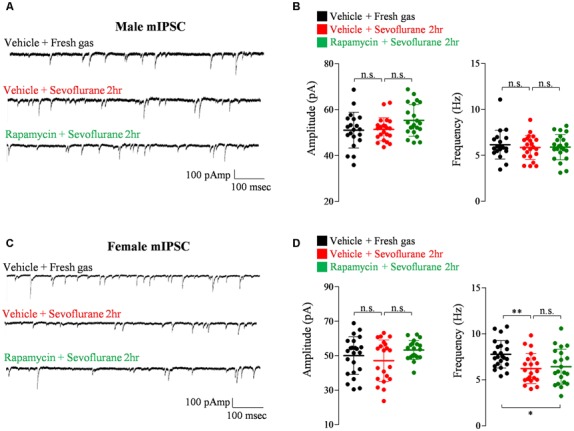
Rapamycin does not prevent the decrease in inhibitory synaptic transmission in PND 16/17 female mice 24 h after sevoflurane exposure. **(A,B)** Frequency and amplitude of miniature inhibitory postsynaptic currents (mIPSCs) in male mice 24 h after sevoflurane exposure with/without rapamycin pretreatment (control: 20 cells from four mice; sevoflurane: 22 cells from four mice; rapamycin: 22 cells from four mice; n.s., not significant). **(C,D)** Frequency and amplitude of mIPSC in female mice 24 h after sevoflurane exposure with/without rapamycin pretreatment (control: 23 cells from four mice; sevoflurane: 21 cells from four mice; rapamycin: 21 cells from four mice; n.s., not significant, *p < 0.05, **p < 0.01).

Exposure of PND 16/17 mice to sevoflurane was shown to induce acute, sex-dependent changes in various brain regions (Chung et al., 2017a; Ju et al., 2019). To extend these findings, we assessed changes of excitatory/inhibitory synaptic transmission in CA1 pyramidal neurons in the hippocampus 24 h after sevoflurane exposure. Sevoflurane increased mEPSC frequency only in male mice (Figure 4), an increase blocked by preinjection of rapamycin (Figures 4A,B). In contrast, sevoflurane affected mIPSC frequency only in female mice (Figure 5), but these changes were unaffected by preinjection of rapamycin (Figures 5C,D). These results suggest that only the sex-dependent changes in excitatory synaptic transmission are mTOR dependent.

## Discussion

Few studies to date have analyzed the mechanisms underlying changes in synaptic transmission after exposure to anesthesia in late postnatal mice. To gain further insight and to identify a possible molecular target for preventing such anesthesia-induced changes, we focused on the mTOR pathway due to the fact that mTOR signaling regulates mitochondrial function (Ramanathan and Schreiber, 2009; Morita et al., 2015, 2017), dendritic spine formation, AMPA receptor synthesis, and excitatory synaptic transmission (Bockaert and Marin, 2015). Our results indicate that the mTOR pathway is associated with the changes that occur after anesthetic exposure in late postnatal male mice.

Neuronal mitochondrial function has been shown to be involved with dendritic spine formation and synaptic transmission (Li et al., 2004; Guo et al., 2017; Rossi and Pekkurnaz, 2019). Since anesthetic exposure increases spinogenesis and induces change in synaptic transmission, we previously suggested the compensatory increase of mitochondria function as a possible mechanism (Chung et al., 2017a). However, we were unable to provide a mechanism by which sevoflurane increased mitochondrial function. Previous studies have suggested that the mTOR complex stimulates the synthesis of mitochondrial components by regulating translation (Morita et al., 2013). In our present study, we also suggest that mTOR regulates sevoflurane-induced changes in mitochondrial function by showing that rapamycin pretreatment efficiently blocks the sevoflurane-induced increases in mitochondrial OCR in both male and female mice. While our results suggest that mTOR may regulate the sevoflurane-induced neurological changes through mitochondrial activation, many studies also show that mTOR signaling itself is an important regulator of dendritic spine formation, AMPA receptor synthesis, and excitatory synaptic transmission (Bockaert and Marin, 2015). Thus, inhibition of the mTOR pathway can prevent the anesthesia-induced changes in a mitochondrial-independent fashion as well. However, considering previous evidence regarding the significance of mitochondrial function with neurological changes, it is highly possible that mTOR regulates the sevoflurane-induced changes through both mitochondrial-dependent and independent pathways.

Sex is recognized as a valuable biological variable in neuroscience (Shansky and Woolley, 2016; Bale and Epperson, 2017; Torres-Rojas and Jones, 2018), and has been shown to affect anesthesia-induced neurotoxicity during neurodevelopment in young animals (Boscolo et al., 2013; Ju et al., 2019). Unfortunately, the majority of clinical studies have been performed with male patients (Lin et al., 2017), and the clinical significance of sex is still in need of further evaluation. Several sex-dependent changes were also discovered in our present study. First, while sevoflurane activates mTOR in both sexes, mTOR-dependent increases in AMPA receptor subunit expression and excitatory synaptic transmission only occurred in male mice. This may be due to sex-dependent differences in the downstream signaling of mTOR. A previous study has shown different downstream activities between male and female mice in non-neural tissues. For instance, when compared to female mice, male mice showed decreased basal mTORC1 activity in the liver and heart tissue, while the basal mTORC2 activity was increased in muscle tissue (Baar et al., 2016). Sevoflurane-induced mTOR activation may result in male-dependent changes due to differences in mTOR downstream signaling in male and female mice. Second, sevoflurane exposure induced different mitochondrial changes between sexes. When measured 24 h after sevoflurane exposure, male mice displayed increases of ADP induced OCR, maximal OCR and ATP production without changes in basal OCR and OXPHOS subunit protein levels. However, all stages of the mitochondrial OCR and OXPHOS subunit protein levels were still increased in female mice. Such discrepancies between male and female mice may be due to the differences in mitochondrial respiratory function, morphology, and reactive oxygen species (ROS) homeostasis (Khalifa et al., 2017). Previous studies also show sex-dependent differences in mitochondrial biogenesis after oxygen-glucose deprivation and reoxygenation (Sharma et al., 2014). Another interesting fact is that sevoflurane-induced activation of the mTOR pathway can sex-dependently affect the expression of a specific set of proteins, as female mice show more significant changes for a mitochondrial protein, but male mice display more difference in an excitatory synaptic protein. While this may be due to distinct downstream mTOR signaling between male and female mice as mentioned above, distinct gene expression (sexually dimorphic genes) and epigenetic sex differences may also be involved (Yang et al., 2006; McCarthy and Nugent, 2015).

An important factor in our study was the duration of rapamycin treatment. Initially, the experiments involved a single injection of rapamycin (Supplementary Figure S1). Although this single injection restored the phosphorylation level of mTOR, it did not prevent the sevoflurane-induced increase in mEPSC frequency in male mice. We next used a daily injection of rapamycin (5 mg/kg/day) for three consecutive days based on previous studies (Zeng et al., 2009; Huang et al., 2010; Hartman et al., 2012). Unlike a single injection, multiple rapamycin injections were capable of preventing the sevoflurane-induced increase in excitatory synaptic transmission. mTOR acts by forming two distinct protein complexes, mTOR Complex 1 (mTORC1) and mTOR Complex 2 (mTORC2). Despite rapamycin being an mTOR inhibitor, prolonged rapamycin treatment is required to block mTORC2. One possible explanation is that blockade of mTORC2 by multiple rapamycin injections is required to block changes in excitatory synaptic transmission.

Another interesting finding of our study is that the female-dependent changes in inhibitory synaptic transmission after sevoflurane exposure seems to be irrelevant of mTOR signaling, as rapamycin pretreatment did not affect inhibitory synaptic transmission in both male and female mice. Also, the changes of inhibitory synaptic transmission observed 24 h after sevoflurane exposure was different than the changes observed shortly after anesthesia induction (Chung et al., 2017a; Ju et al., 2019). Whereas mIPSC frequency increased in female mice 6 h after sevoflurane exposure (Ju et al., 2019), mIPSC frequency decreased 24 h after exposure. Genetic mouse models of neurodevelopmental disorders have also shown reversed changes in synaptic transmission during development (Chung et al., 2019). Although these reversed changes may be due to compensation mechanisms, more studies focusing on the mechanism behind time-dependent changes in inhibitory synaptic transmission in female mice are required.

This study has several limitations. Although the oxygen concentration during anesthesia was relatively low (FiO_2_ 0.4), thereby avoiding oxygen toxicity, we are unable to rule out the effects of slight changes in arterial carbon dioxide (PaCO_2_) and blood pH after exposure to sevoflurane for 2 h (Chung et al., 2017a). Another limitation was the inconsistency of brain regions among experiments. Western blot and mitochondrial experiments were performed using whole-brain samples, enabling us to confirm widespread changes in mTOR signaling and mitochondrial function. However, electrophysiology experiments were performed using only hippocampal neurons. We have previously reported that sevoflurane exposure induces similar, but slightly different, changes in different brain regions (Chung et al., 2017a; Ju et al., 2019). Since the importance of sex regarding sevoflurane-induced changes were more thoroughly addressed in the hippocampus, we evaluated the possible sex-dependent effects of rapamycin in the hippocampus (CA1 region).

In conclusion, exposure of PND 16/17 mice to sevoflurane induces mTOR phosphorylation, leading to enhanced mitochondrial function and male-dependent excitatory synaptic transmission. Although further studies regarding the mechanism behind inhibitory synaptic changes in female mice are necessary, our results suggest that mTOR inhibitors may be potential therapeutic agents for anesthesia-induced changes during neurodevelopment.

## Data Availability Statement

All datasets generated for this study are included in the article/Supplementary Material.

## Ethics Statement

The animal study was reviewed and approved by Committees of Chungnam National University, Daejeon, South Korea (CNU-01135). Written informed consent was obtained from the owners for the participation of their animals in this study.

## Author Contributions

XJ and MR: helped design, development, execution of experiments and preparation of manuscript. SP and BH: helped perform the statistics. JC, YL and SY: helped execution of experiments. WL, YS, S-HY, GK, YHK and YK: helped design and development of experiments, oversight. WC and JH: helped design, development and execution of experiments, oversight, preparation of manuscript.

## Conflict of Interest

The authors declare that the research was conducted in the absence of any commercial or financial relationships that could be construed as a potential conflict of interest.
