# Supplementary material for: The mTOR Inhibitor Rapamycin Prevents General Anesthesia-Induced Changes in Synaptic Transmission and Mitochondrial Respiration in Late Postnatal Mice
Source: Front Cell Neurosci. 2020 Jan 28;14:4. doi: 10.3389/fncel.2020.00004 (PMC6997293; doi:10.3389/fncel.2020.00004)
Supplement: Supplementary file 1 [file Data_Sheet_1.PDF]

Fig1_male mTor ratio Data analysis using R

By Sangil Park & Boohwi Hong

1 Package install

```
Packages <- c("tidyverse", "car", "dunn.test","onewaytests","FSA")
lapply(Packages, library, character.only = TRUE)
```

2 Data import

```
d1<- read.csv("/Users/koho0/Desktop/stats/fig1_male mTor ratio.csv")
```

3 Data structure

```
str(d1)
```

```
## 'data.frame':    14 obs. of  3 variables:
##  $ subject: int  1 2 3 4 5 6 7 8 9 10 ...
##  $ group  : Factor w/ 3 levels "rapamycin+sevoflurane",..: 2 2 2 2 2 3 3 3 3 1 ...
##  $ ratio  : num  1.119 1.499 0.567 0.832 0.983 ...
```

4 Explorative data analysis with graphics

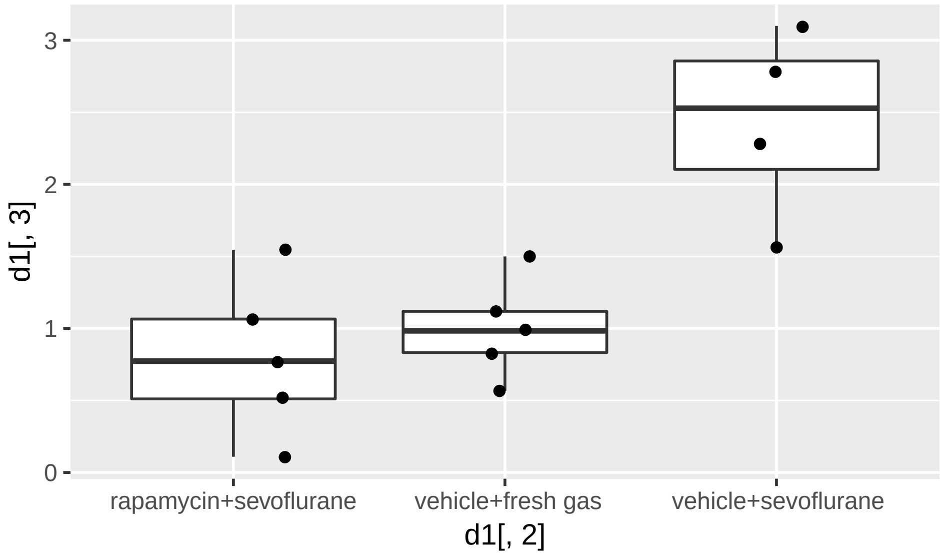

5 Easystat function developed by S. Park (available at https://rpubs.com/goodlebang)

6 Statistical Result

```r
easystat(d1)
```

```
## 1. Normality assumption test by Shapiro_Wilk test is
##  p = 0.959
##  Normality assumption was not rejected
## 2. Equal variance test by Bartlett test is
##  p = 0.518
##  Equal variance assumption was not rejected
## 3. The result of anova is
##  p = 0.001
##  A statistically significant difference exist between groups
```

```
##   Tukey multiple comparisons of means
##     95% family-wise confidence level
##
## Fit: aov(formula = d1[, 3] ~ d1[, 2], data = d1)
##
## $`d1[, 2]`
##                                                diff        lwr      upr
## vehicle+fresh gas-rapamycin+sevoflurane   0.1993408 -0.6921752 1.090857
## vehicle+sevoflurane-rapamycin+sevoflurane 1.6312303  0.6856349 2.576826
## vehicle+sevoflurane-vehicle+fresh gas     1.4318896  0.4862941 2.377485
##                                               p adj
## vehicle+fresh gas-rapamycin+sevoflurane   0.8209340
## vehicle+sevoflurane-rapamycin+sevoflurane 0.0018394
## vehicle+sevoflurane-vehicle+fresh gas     0.0046701
```

Fig1_female mTor ratio Data analysis using R

By Sangil Park & Boohwi Hong

1 Package install

```
Packages <- c("tidyverse", "car", "dunn.test","onewaytests","FSA")
lapply(Packages, library, character.only = TRUE)
```

2 Data import

```
d1<- read.csv("/Users/koho0/Desktop/stats/fig1_female mTor ratio.csv")
```

3 Data structure

```
str(d1)
```

```
## 'data.frame':    14 obs. of  3 variables:
##  $ subject: int  1 2 3 4 5 6 7 8 9 10 ...
##  $ group  : Factor w/ 3 levels "rapamycin+sevoflurane",..: 2 2 2 2 3 3 3 3 3 1 ...
##  $ ratio  : num  0.739 0.823 1.049 1.39 2.365 ...
```

4 Explorative data analysis with graphics

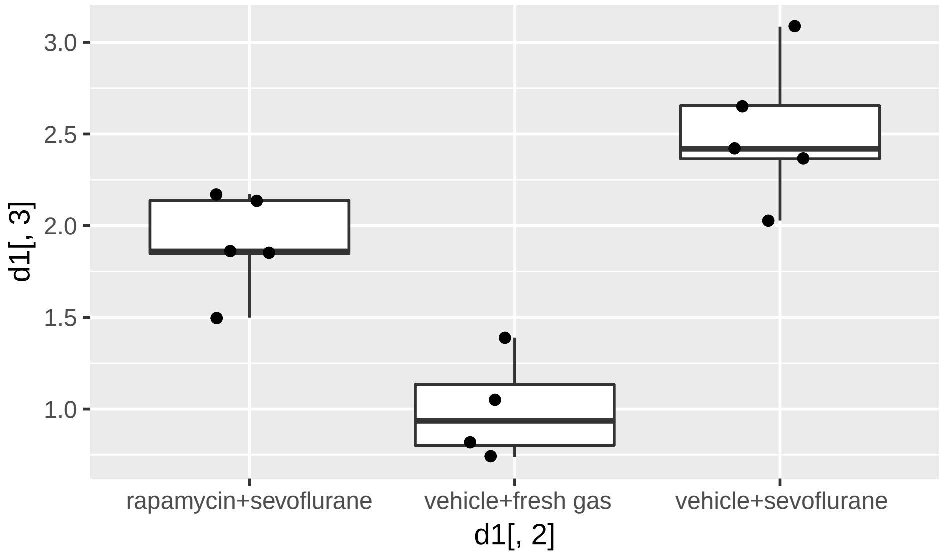

5 Easystat function developed by S. Park (available at https://rpubs.com/goodlebang)

6 Statistical Result

```r
easystat(d1)
```

```
## 1. Normality assumption test by Shapiro_Wilk test is
##  p = 0.991
##  Normality assumption was not rejected
## 2. Equal variance test by Bartlett test is
##  p = 0.761
##  Equal variance assumption was not rejected
## 3. The result of anova is
##  p = 0.0001
##  A statistically significant difference exist between groups
```

```
##   Tukey multiple comparisons of means
##     95% family-wise confidence level
##
## Fit: aov(formula = d1[, 3] ~ d1[, 2], data = d1)
##
## $`d1[, 2]`
##                                                diff         lwr
## vehicle+fresh gas-rapamycin+sevoflurane   -0.9030379 -1.49221329
## vehicle+sevoflurane-rapamycin+sevoflurane  0.6073292  0.05184935
## vehicle+sevoflurane-vehicle+fresh gas      1.5103671  0.92119175
##                                                 upr     p adj
## vehicle+fresh gas-rapamycin+sevoflurane   -0.3138625 0.0042983
## vehicle+sevoflurane-rapamycin+sevoflurane  1.1628091 0.0324820
## vehicle+sevoflurane-vehicle+fresh gas      2.0995425 0.0000684
```
