# Supplementary material for: The mTOR Inhibitor Rapamycin Prevents General Anesthesia-Induced Changes in Synaptic Transmission and Mitochondrial Respiration in Late Postnatal Mice
Source: Front Cell Neurosci. 2020 Jan 28;14:4. doi: 10.3389/fncel.2020.00004 (PMC6997293; doi:10.3389/fncel.2020.00004)
Supplement: Supplementary file 2 [file Data_Sheet_2.PDF]

Fig2_male NDUFB8 Data analysis using R

By Sangil Park & Boohwi Hong

1 Package install

```
Packages <- c("tidyverse", "car", "dunn.test","onewaytests","FSA")
lapply(Packages, library, character.only = TRUE)
```

2 Data import

```
d1<- read.csv("/Users/koho0/Desktop/Fig2_male ND1_.csv")
```

3 Data structure

```
str(d1)
```

```
## 'data.frame':    14 obs. of  3 variables:
##  $ subject: int  1 2 3 4 5 6 7 8 9 10 ...
##  $ group  : Factor w/ 3 levels "rapamycin+sevoflurane",..: 2 2 2 2 2 3 3 3 3 1 ...
##  $ ND1    : num  1.101 0.813 1.058 1.089 0.94 ...
```

4 Explorative data analysis with graphics

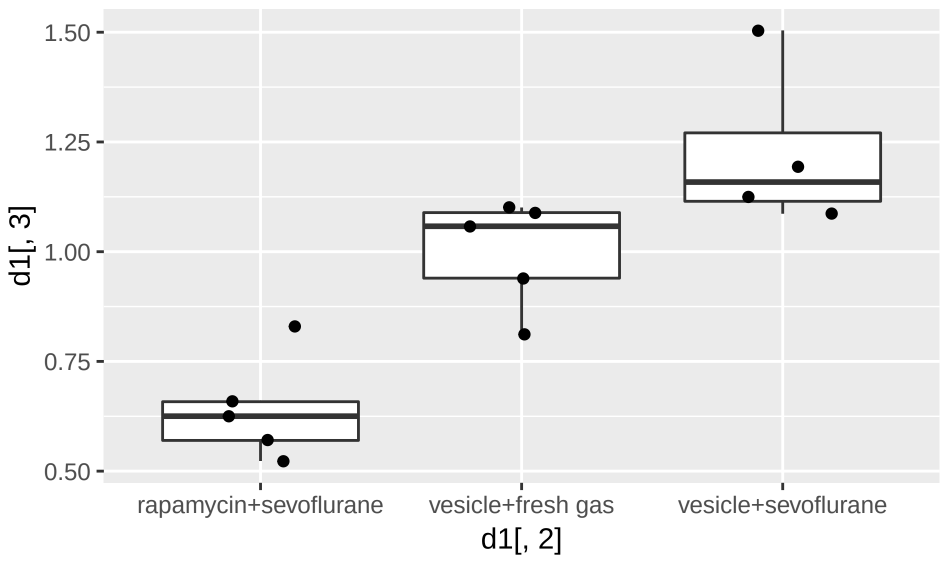

5 Easystat function developed by S. Park (available at https://rpubs.com/goodlebang)

6 Statistical Result

```r
easystat(d1)
```

```
## 1. Normality assumption test by Shapiro_Wilk test is
##  p = 0.739
##  Normality assumption was not rejected
## 2. Equal variance test by Bartlett test is
##  p = 0.632
##  Equal variance assumption was not rejected
## 3. The result of anova is
##  p = 0.0002
##  A statistically significant difference exist between groups
```

```
##   Tukey multiple comparisons of means
##     95% family-wise confidence level
##
## Fit: aov(formula = d1[, 3] ~ d1[, 2], data = d1)
##
## $`d1[, 2]`
##                                               diff         lwr       upr
## vesicle+fresh gas-rapamycin+sevoflurane   0.3585919  0.11487840 0.6023054
## vesicle+sevoflurane-rapamycin+sevoflurane 0.5855507  0.32705349 0.8440479
## vesicle+sevoflurane-vesicle+fresh gas     0.2269588 -0.03153842 0.4854560
##                                              p adj
## vesicle+fresh gas-rapamycin+sevoflurane   0.0056696
## vesicle+sevoflurane-rapamycin+sevoflurane 0.0002041
## vesicle+sevoflurane-vesicle+fresh gas     0.0869916
```

Fig2_male COX4 Data analysis using R

By Sangil Park & Boohwi Hong

1 Package install

```
Packages <- c("tidyverse", "car", "dunn.test","onewaytests","FSA")
lapply(Packages, library, character.only = TRUE)
```

2 Data import

```
d1<- read.csv("/Users/koho0/Desktop/Fig2_male COX4_.csv")
```

3 Data structure

```
str(d1)
```

```
## 'data.frame':    14 obs. of  3 variables:
##  $ subject: int  1 2 3 4 5 6 7 8 9 10 ...
##  $ group  : Factor w/ 3 levels "rapamycin+sevoflurane",..: 2 2 2 2 2 3 3 3 3 1 ...
##  $ COX4   : num  1.344 0.979 1.11 0.767 0.8 ...
```

4 Explorative data analysis with graphics

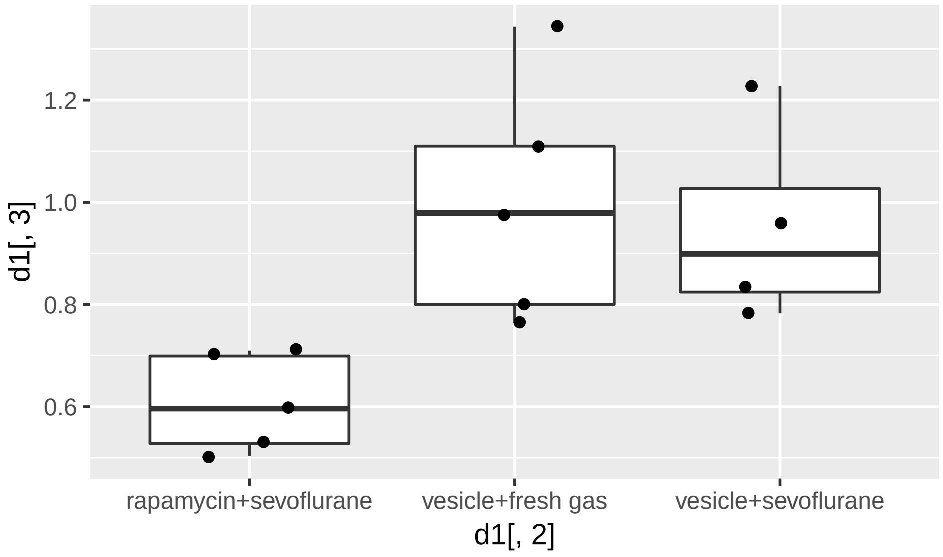

5 Easystat function developed by S. Park (available at https://rpubs.com/goodlebang)

6 Statistical Result

```r
easystat(d1)
```

```
## 1. Normality assumption test by Shapiro_Wilk test is
##  p = 0.528
##  Normality assumption was not rejected
## 2. Equal variance test by Bartlett test is
##  p = 0.264
##  Equal variance assumption was not rejected
## 3. The result of anova is
##  p = 0.0136
##  A statistically significant difference exist between groups
```

```
##   Tukey multiple comparisons of means
##     95% family-wise confidence level
##
## Fit: aov(formula = d1[, 3] ~ d1[, 2], data = d1)
##
## $`d1[, 2]`
##                                                   diff          lwr
## vesicle+fresh gas-rapamycin+sevoflurane    0.39267006  0.075740551
## vesicle+sevoflurane-rapamycin+sevoflurane  0.34484802  0.008693512
## vesicle+sevoflurane-vesicle+fresh gas     -0.04782204 -0.383976543
##                                                 upr     p adj
## vesicle+fresh gas-rapamycin+sevoflurane   0.7095996 0.0165060
## vesicle+sevoflurane-rapamycin+sevoflurane 0.6810025 0.0443878
## vesicle+sevoflurane-vesicle+fresh gas     0.2883325 0.9224072
```

Fig2_female NDUFB8 Data analysis using R

By Sangil Park & Boohwi Hong

1 Package install

```
Packages <- c("tidyverse", "car", "dunn.test","onewaytests","FSA")
lapply(Packages, library, character.only = TRUE)
```

2 Data import

```
d1<- read.csv("/Users/koho0/Desktop/stats/fig2_female ND1.csv")
```

3 Data structure

```
str(d1)
```

```
## 'data.frame':    14 obs. of  3 variables:
##  $ subject: int  1 2 3 4 5 6 7 8 9 10 ...
##  $ group  : Factor w/ 3 levels "rapamycin+sevoflurane",..: 2 2 2 2 3 3 3 3 3 1 ...
##  $ ND1    : num  1.055 0.947 1.053 0.945 1.587 ...
```

4 Explorative data analysis with graphics

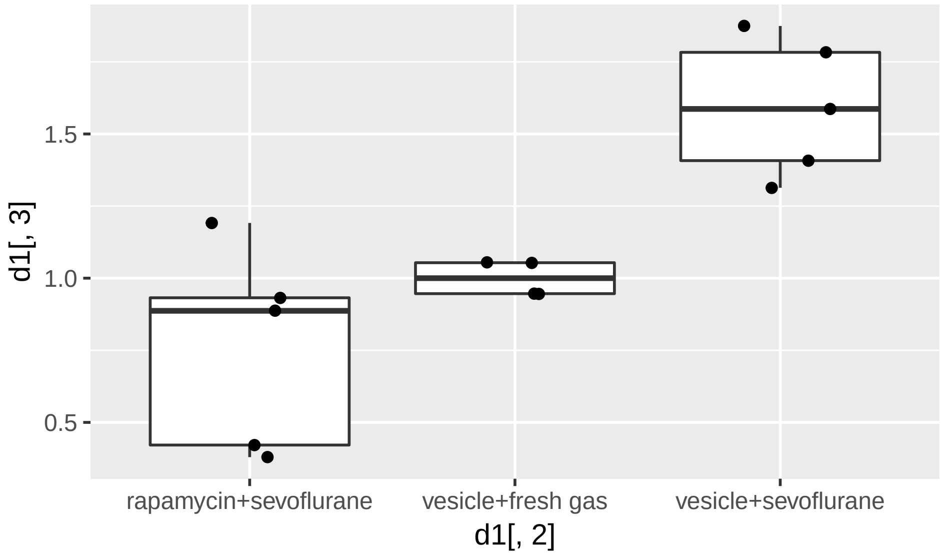

5 Easystat function developed by S. Park (available at https://rpubs.com/goodlebang)

6 Statistical Result

```r
easystat(d1)
```

```
## 1. Normality assumption test by Shapiro_Wilk test is
##  p = 0.929
##  Normality assumption was not rejected
## 2. Equal variance test by Bartlett test is
##  p = 0.051
##  Equal variance assumption was not rejected
## 3. The result of anova is
##  p = 0.0010
##  A statistically significant difference exist between groups
```

```
##   Tukey multiple comparisons of means
##     95% family-wise confidence level
##
## Fit: aov(formula = d1[, 3] ~ d1[, 2], data = d1)
##
## $`d1[, 2]`
##                                                diff        lwr       upr
## vesicle+fresh gas-rapamycin+sevoflurane   0.2377703 -0.2291810 0.7047215
## vesicle+sevoflurane-rapamycin+sevoflurane 0.8307190  0.3904731 1.2709648
## vesicle+sevoflurane-vesicle+fresh gas     0.5929487  0.1259975 1.0599000
##                                               p adj
## vesicle+fresh gas-rapamycin+sevoflurane   0.3861715
## vesicle+sevoflurane-rapamycin+sevoflurane 0.0009240
## vesicle+sevoflurane-vesicle+fresh gas     0.0143046
```

Fig2_female COX4 Data analysis using R

By Sangil Park & Boohwi Hong

1 Package install

```
Packages <- c("tidyverse", "car", "dunn.test","onewaytests","FSA")
lapply(Packages, library, character.only = TRUE)
```

2 Data import

```
d1<- read.csv("/Users/koho0/Desktop/stats/fig2_female COX4.csv")
```

3 Data structure

```
str(d1)
```

```
## 'data.frame':    14 obs. of  3 variables:
##  $ subject: int  1 2 3 4 5 6 7 8 9 10 ...
##  $ group  : Factor w/ 3 levels "rapamycin+sevoflurane",..: 2 2 2 2 3 3 3 3 3 1 ...
##  $ COX4   : num  1.01 0.8 1.11 1.09 1.61 ...
```

4 Explorative data analysis with graphics

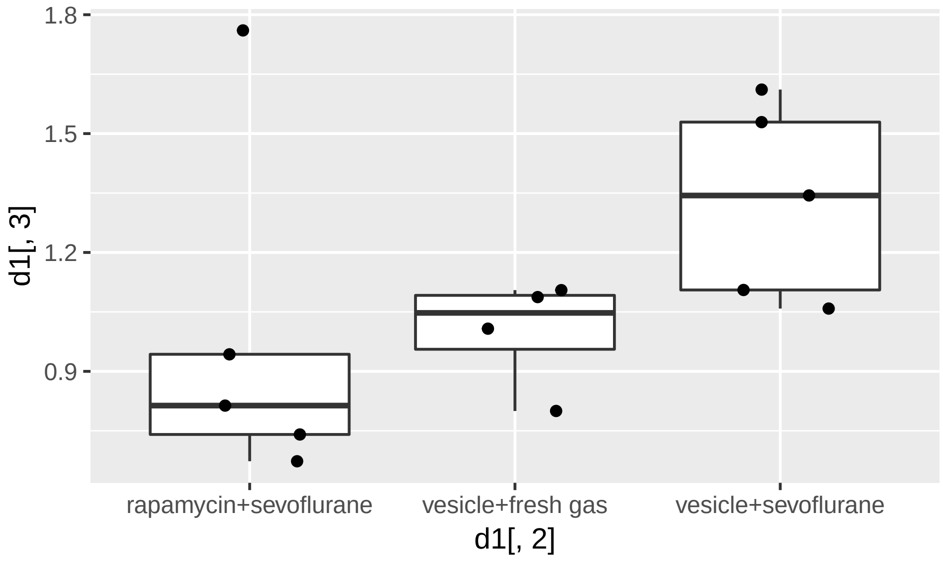

5 Easystat function developed by S. Park (available at https://rpubs.com/goodlebang)

6 Statistical Result

```
easystat(d1)
```

```
## 1. Normality assumption test by Shapiro_Wilk test is
##  p = 0.04
##  Normality assumption was rejected
## 2. The result of Kruskall_Wallis test:
##  p = 0.106
##  A statistically significant difference do not exist between groups
```

Fig2_male OCR_stage I Data analysis using R

By Sangil Park & Boohwi Hong

1 Package install

```
Packages <- c("tidyverse", "car", "dunn.test","onewaytests","FSA")
lapply(Packages, library, character.only = TRUE)
```

2 Data import

```
d1<- read.csv("/Users/koho0/Desktop/Fig2_male OCR_stage I.csv")
```

3 Data structure

```
str(d1)
```

```
## 'data.frame':    39 obs. of  4 variables:
##  $ subject: int  1 2 3 4 5 6 7 8 9 10 ...
##  $ group  : Factor w/ 3 levels "rapamycin+sevoflurane",..: 2 2 2 2 2 2 2 2 2 2 ...
##  $ OCR    : int  57 116 90 84 50 96 84 75 65 95 ...
##  $ X      : logi  NA NA NA NA NA NA ...
```

4 Explorative data analysis with graphics

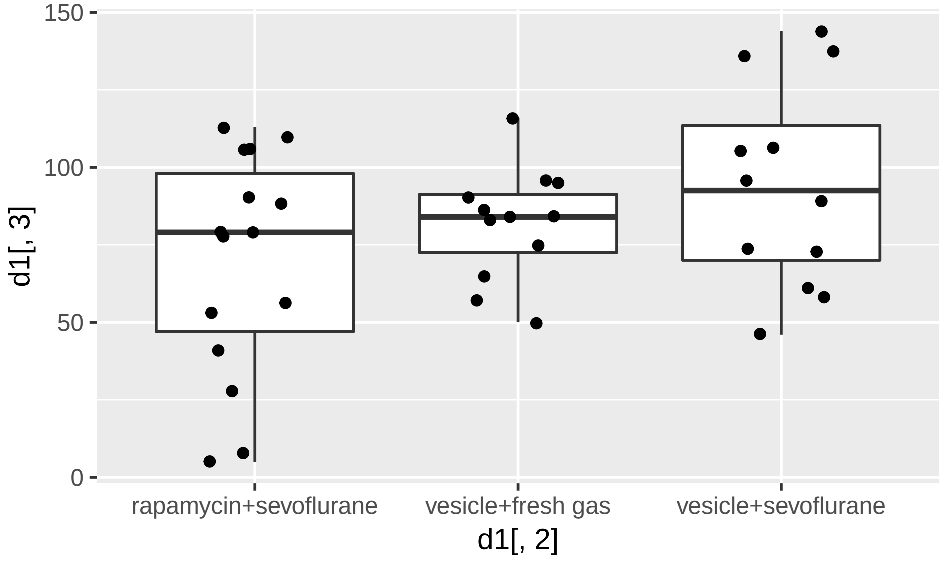

5 Easystat function developed by S. Park (available at https://rpubs.com/goodlebang)

6 Statistical Result

```r
easystat(d1)
```

```
## 1. Normality assumption test by Shapiro_Wilk test is
##  p = 0.355
##  Normality assumption was not rejected
## 2. Equal variance test by Bartlett test is
##  p = 0.075
##  Equal variance assumption was not rejected
## 3. The result of anova is
##  p = 0.1329
##  A statistically significant difference do not exist between groups
```

Fig2_male OCR_stage II Data analysis using R

By Sangil Park & Boohwi Hong

1 Package install

```
Packages <- c("tidyverse", "car", "dunn.test","onewaytests","FSA")
lapply(Packages, library, character.only = TRUE)
```

2 Data import

```
d1<- read.csv("/Users/koho0/Desktop/Fig2_male OCR_stage II.csv")
```

3 Data structure

```
str(d1)
```

```
## 'data.frame':    39 obs. of  3 variables:
##  $ subject: int  1 2 3 4 5 6 7 8 9 10 ...
##  $ group  : Factor w/ 3 levels "rapamycin+sevoflurane",..: 2 2 2 2 2 2 2 2 2 2 ...
##  $ OCR    : int  188 171 243 170 192 174 243 171 169 167 ...
```

4 Explorative data analysis with graphics

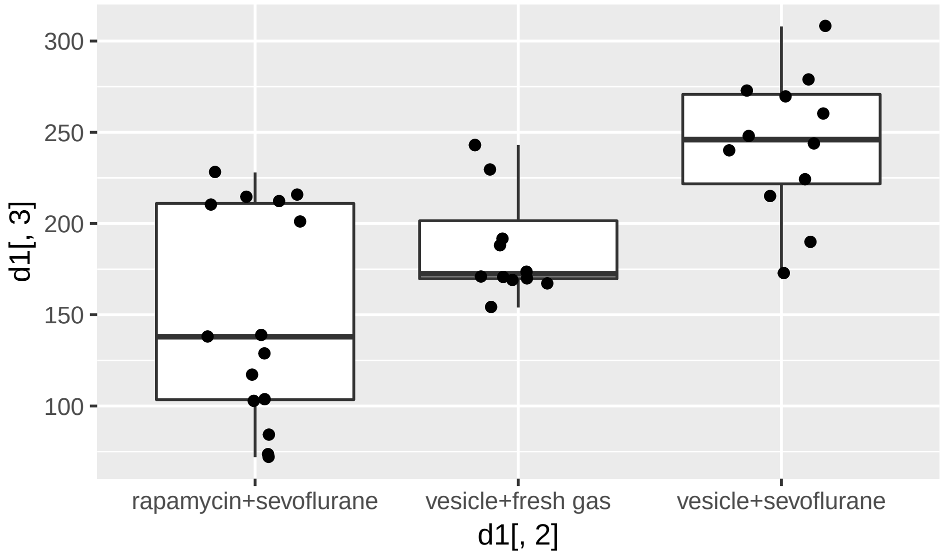

5 Easystat function developed by S. Park (available at https://rpubs.com/goodlebang)

6 Statistical Result

```r
easystat(d1)
```

```
## 1. Normality assumption test by Shapiro_Wilk test is
##  p = 0.09
##  Normality assumption was not rejected
## 2. Equal variance test by Bartlett test is
##  p = 0.095
##  Equal variance assumption was not rejected
## 3. The result of anova is
##  p = 0.0000
##  A statistically significant difference exist between groups
```

```
##   Tukey multiple comparisons of means
##     95% family-wise confidence level
##
## Fit: aov(formula = d1[, 3] ~ d1[, 2], data = d1)
##
## $`d1[, 2]`
##                                              diff       lwr       upr
## vesicle+fresh gas-rapamycin+sevoflurane   39.86667 -3.120159  82.85349
## vesicle+sevoflurane-rapamycin+sevoflurane 94.20000 51.213175 137.18683
## vesicle+sevoflurane-vesicle+fresh gas     54.33333  9.021241  99.64543
##                                              p adj
## vesicle+fresh gas-rapamycin+sevoflurane   0.0736561
## vesicle+sevoflurane-rapamycin+sevoflurane 0.0000148
## vesicle+sevoflurane-vesicle+fresh gas     0.0157190
```

Fig2_male OCR_stage III Data analysis using R

By Sangil Park & Boohwi Hong

1 Package install

```
Packages <- c("tidyverse", "car", "dunn.test","onewaytests","FSA")
lapply(Packages, library, character.only = TRUE)
```

2 Data import

```
d1<- read.csv("/Users/koho0/Desktop/Fig2_male OCR_stage III.csv")
```

3 Data structure

```
str(d1)
```

```
## 'data.frame':    39 obs. of  3 variables:
##  $ subject: int  1 2 3 4 5 6 7 8 9 10 ...
##  $ group  : Factor w/ 3 levels "rapamycin+sevoflurane",..: 2 2 2 2 2 2 2 2 2 2 ...
##  $ OCR    : int  28 28 37 31 32 39 50 39 41 41 ...
```

4 Explorative data analysis with graphics

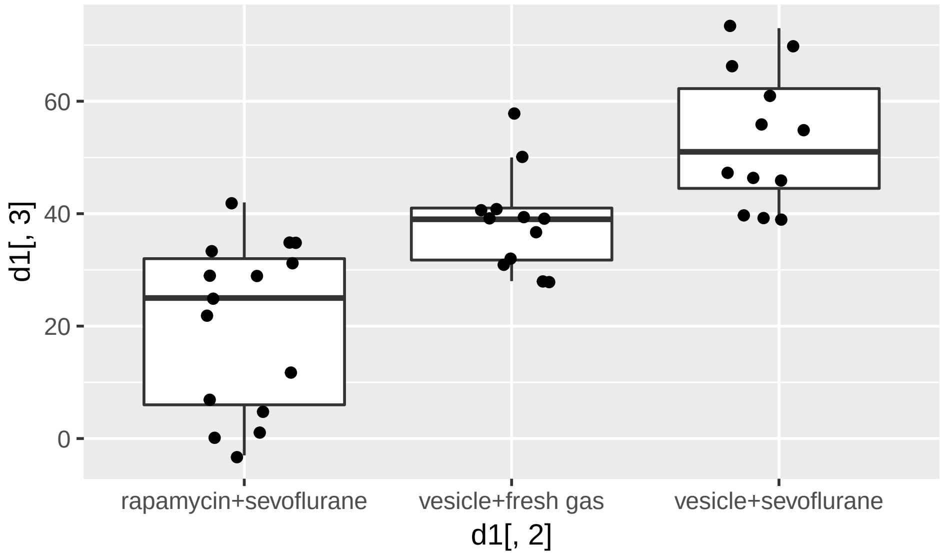

5 Easystat function developed by S. Park (available at https://rpubs.com/goodlebang)

6 Statistical Result

```r
easystat(d1)
```

```
## 1. Normality assumption test by Shapiro_Wilk test is
##  p = 0.37
##  Normality assumption was not rejected
## 2. Equal variance test by Bartlett test is
##  p = 0.204
##  Equal variance assumption was not rejected
## 3. The result of anova is
##  p = 0.0000
##  A statistically significant difference exist between groups
```

```
##   Tukey multiple comparisons of means
##     95% family-wise confidence level
##
## Fit: aov(formula = d1[, 3] ~ d1[, 2], data = d1)
##
## $`d1[, 2]`
##                                              diff       lwr      upr
## vesicle+fresh gas-rapamycin+sevoflurane   18.38333  6.535232 30.23144
## vesicle+sevoflurane-rapamycin+sevoflurane 32.96667 21.118565 44.81477
## vesicle+sevoflurane-vesicle+fresh gas     14.58333  2.094337 27.07233
##                                              p adj
## vesicle+fresh gas-rapamycin+sevoflurane   0.0015581
## vesicle+sevoflurane-rapamycin+sevoflurane 0.0000002
## vesicle+sevoflurane-vesicle+fresh gas     0.0190244
```

Fig2_male OCR_stage IV Data analysis using R

By Sangil Park & Boohwi Hong

1 Package install

```
Packages <- c("tidyverse", "car", "dunn.test","onewaytests","FSA")
lapply(Packages, library, character.only = TRUE)
```

2 Data import

```
d1<- read.csv("/Users/koho0/Desktop/Fig2_male OCR_stage IV.csv")
```

3 Data structure

```
str(d1)
```

```
## 'data.frame':    39 obs. of  3 variables:
##  $ subject: int  1 2 3 4 5 6 7 8 9 10 ...
##  $ group  : Factor w/ 3 levels "rapamycin+sevoflurane",..: 2 2 2 2 2 2 2 2 2 2 ...
##  $ OCR    : int  69 78 117 73 72 84 111 75 72 82 ...
```

4 Explorative data analysis with graphics

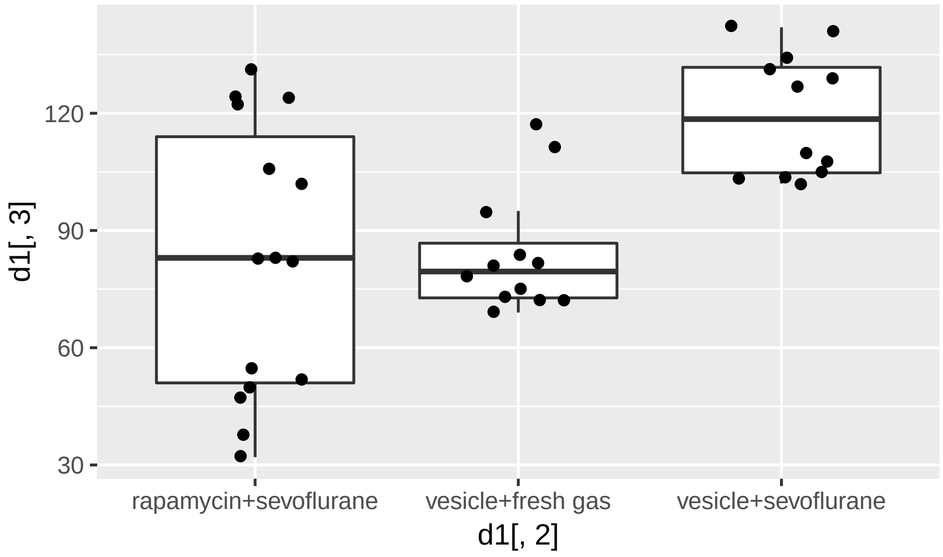

5 Easystat function developed by S. Park (available at https://rpubs.com/goodlebang)

6 Statistical Result

```r
easystat(d1)
```

```
## 1. Normality assumption test by Shapiro_Wilk test is
##  p = 0.713
##  Normality assumption was not rejected
## 2. Equal variance test by Bartlett test is
##  p = 0.005
##  Equal variance assumption was rejected
## 3. The result of Welch ANOVA is
##  p = 0.000
##  A statistically significant difference exist between groups
```

```
##   Tukey multiple comparisons of means
##     95% family-wise confidence level
##
## Fit: aov(formula = d1[, 3] ~ d1[, 2])
##
## $`d1[, 2]`
##                                              diff       lwr      upr
## vesicle+fresh gas-rapamycin+sevoflurane    2.016667 -21.50904 25.54237
## vesicle+sevoflurane-rapamycin+sevoflurane 37.600000  14.07429 61.12571
## vesicle+sevoflurane-vesicle+fresh gas     35.583333  10.78506 60.38161
##                                               p adj
## vesicle+fresh gas-rapamycin+sevoflurane   0.9761040
## vesicle+sevoflurane-rapamycin+sevoflurane 0.0011261
## vesicle+sevoflurane-vesicle+fresh gas     0.0034506
```

Fig2_female OCR_stage I Data analysis using R

By Sangil Park & Boohwi Hong

1 Package install

```
Packages <- c("tidyverse", "car", "dunn.test","onewaytests","FSA")
lapply(Packages, library, character.only = TRUE)
```

2 Data import

```
d1<- read.csv("/Users/koho0/Desktop/Fig2_female OCR_stage I.csv")
```

3 Data structure

```
str(d1)
```

```
## 'data.frame':    36 obs. of  3 variables:
##  $ subject: int  1 2 3 4 5 6 7 8 9 10 ...
##  $ group  : Factor w/ 3 levels "rapamycin+sevoflurane",..: 2 2 2 2 2 2 2 2 2 2 ...
##  $ OCR    : int  72 25 58 64 65 48 124 74 65 67 ...
```

4 Explorative data analysis with graphics

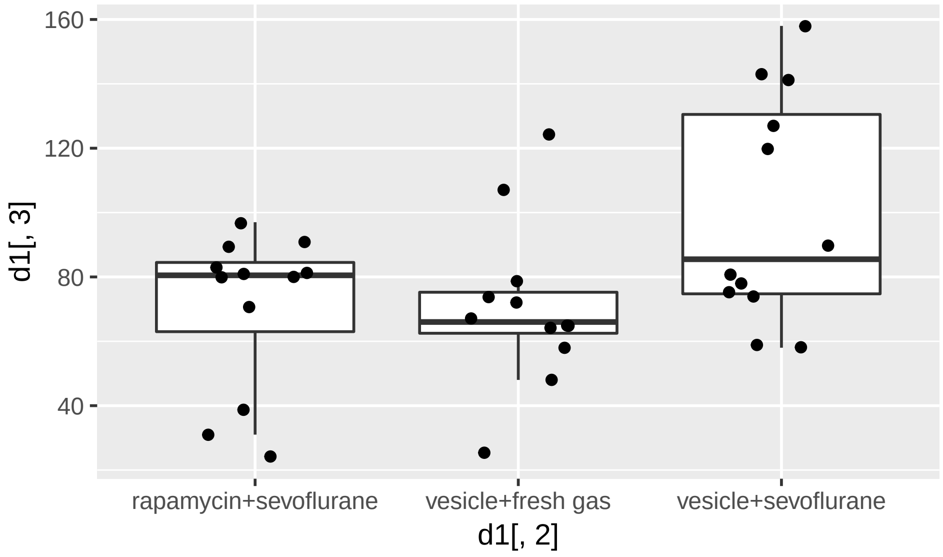

5 Easystat function developed by S. Park (available at https://rpubs.com/goodlebang)

6 Statistical Result

```r
easystat(d1)
```

```
## 1. Normality assumption test by Shapiro_Wilk test is
##  p = 0.566
##  Normality assumption was not rejected
## 2. Equal variance test by Bartlett test is
##  p = 0.418
##  Equal variance assumption was not rejected
## 3. The result of anova is
##  p = 0.023
##  A statistically significant difference exist between groups
```

```
##   Tukey multiple comparisons of means
##     95% family-wise confidence level
##
## Fit: aov(formula = d1[, 3] ~ d1[, 2], data = d1)
##
## $`d1[, 2]`
##                                                 diff         lwr      upr
## vesicle+fresh gas-rapamycin+sevoflurane    0.08333333 -28.8760260 29.04269
## vesicle+sevoflurane-rapamycin+sevoflurane 29.75000000   0.7906407 58.70936
## vesicle+sevoflurane-vesicle+fresh gas     29.66666667   0.7073074 58.62603
##                                               p adj
## vesicle+fresh gas-rapamycin+sevoflurane   0.9999725
## vesicle+sevoflurane-rapamycin+sevoflurane 0.0430499
## vesicle+sevoflurane-vesicle+fresh gas     0.0437397
```

Fig2_female OCR_stage II Data analysis using R

By Sangil Park & Boohwi Hong

1 Package install

```
Packages <- c("tidyverse", "car", "dunn.test","onewaytests","FSA")
lapply(Packages, library, character.only = TRUE)
```

2 Data import

```
d1<- read.csv("/Users/koho0/Desktop/Fig2_female OCR_stage II.csv")
```

3 Data structure

```
str(d1)
```

```
## 'data.frame':    36 obs. of  3 variables:
##  $ subject: int  1 2 3 4 5 6 7 8 9 10 ...
##  $ group  : Factor w/ 3 levels "rapamycin+sevoflurane",..: 2 2 2 2 2 2 2 2 2 2 ...
##  $ OCR    : int  108 156 156 153 131 163 175 158 134 152 ...
```

4 Explorative data analysis with graphics

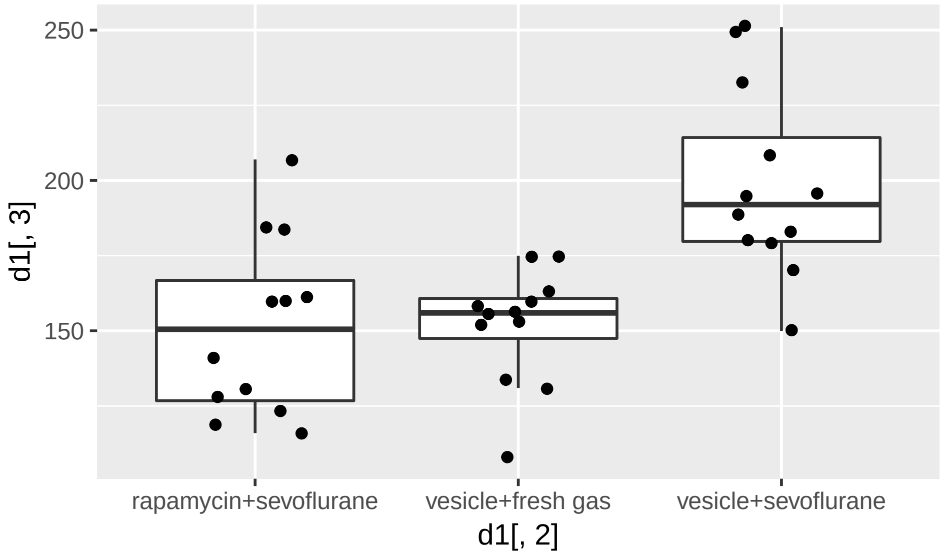

5 Easystat function developed by S. Park (available at https://rpubs.com/goodlebang)

6 Statistical Result

```r
easystat(d1)
```

```
## 1. Normality assumption test by Shapiro_Wilk test is
##  p = 0.46
##  Normality assumption was not rejected
## 2. Equal variance test by Bartlett test is
##  p = 0.253
##  Equal variance assumption was not rejected
## 3. The result of anova is
##  p = 0.0001
##  A statistically significant difference exist between groups
```

```
##   Tukey multiple comparisons of means
##     95% family-wise confidence level
##
## Fit: aov(formula = d1[, 3] ~ d1[, 2], data = d1)
##
## $`d1[, 2]`
##                                              diff       lwr      upr
## vesicle+fresh gas-rapamycin+sevoflurane    0.5833333 -26.69688 27.86354
## vesicle+sevoflurane-rapamycin+sevoflurane 47.4166667  20.13646 74.69688
## vesicle+sevoflurane-vesicle+fresh gas     46.8333333  19.55312 74.11354
##                                             p adj
## vesicle+fresh gas-rapamycin+sevoflurane   0.9984834
## vesicle+sevoflurane-rapamycin+sevoflurane 0.0004523
## vesicle+sevoflurane-vesicle+fresh gas     0.0005252
```

Fig2_female OCR_stage III Data analysis using R

By Sangil Park & Boohwi Hong

1 Package install

```
Packages <- c("tidyverse", "car", "dunn.test","onewaytests","FSA")
lapply(Packages, library, character.only = TRUE)
```

2 Data import

```
d1<- read.csv("/Users/koho0/Desktop/Fig2_female OCR_stage III.csv")
```

3 Data structure

```
str(d1)
```

```
## 'data.frame':    36 obs. of  3 variables:
##  $ subject: int  1 2 3 4 5 6 7 8 9 10 ...
##  $ group  : Factor w/ 3 levels "rapamycin+sevoflurane",..: 2 2 2 2 2 2 2 2 2 2 ...
##  $ OCR    : int  -1 30 38 14 13 30 48 15 26 45 ...
```

4 Explorative data analysis with graphics

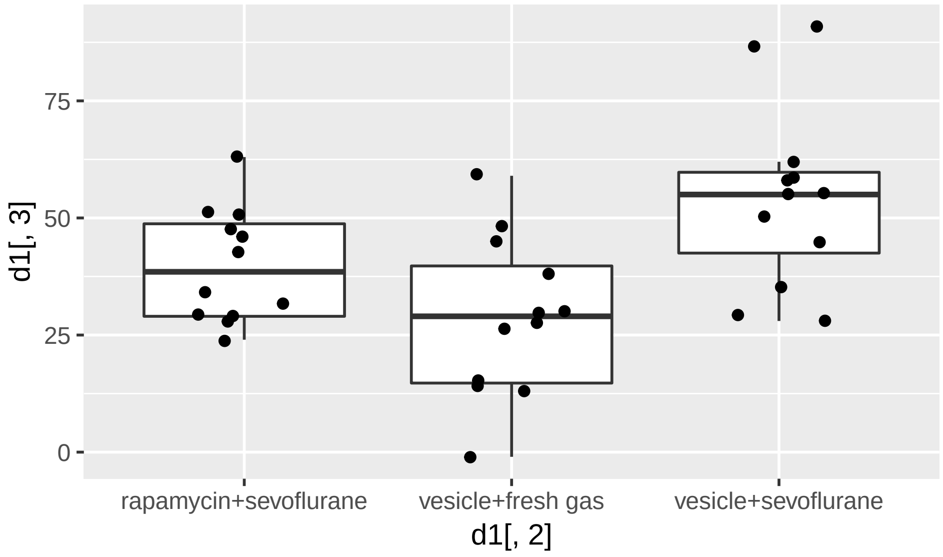

5 Easystat function developed by S. Park (available at https://rpubs.com/goodlebang)

6 Statistical Result

```r
easystat(d1)
```

```
## 1. Normality assumption test by Shapiro_Wilk test is
##  p = 0.675
##  Normality assumption was not rejected
## 2. Equal variance test by Bartlett test is
##  p = 0.292
##  Equal variance assumption was not rejected
## 3. The result of anova is
##  p = 0.0025
##  A statistically significant difference exist between groups
```

```
##   Tukey multiple comparisons of means
##     95% family-wise confidence level
##
## Fit: aov(formula = d1[, 3] ~ d1[, 2], data = d1)
##
## $`d1[, 2]`
##                                                diff        lwr      upr
## vesicle+fresh gas-rapamycin+sevoflurane   -11.08333 -27.744876  5.57821
## vesicle+sevoflurane-rapamycin+sevoflurane  14.66667  -1.994876 31.32821
## vesicle+sevoflurane-vesicle+fresh gas      25.75000   9.088457 42.41154
##                                               p adj
## vesicle+fresh gas-rapamycin+sevoflurane   0.2465908
## vesicle+sevoflurane-rapamycin+sevoflurane 0.0933460
## vesicle+sevoflurane-vesicle+fresh gas     0.0017064
```

Fig2_female OCR_stage IV Data analysis using R

By Sangil Park & Boohwi Hong

1 Package install

```
Packages <- c("tidyverse", "car", "dunn.test","onewaytests","FSA")
lapply(Packages, library, character.only = TRUE)
```

2 Data import

```
d1<- read.csv("/Users/koho0/Desktop/Fig2_female OCR_stage IV.csv")
```

3 Data structure

```
str(d1)
```

```
## 'data.frame':    36 obs. of  3 variables:
##  $ subject: int  1 2 3 4 5 6 7 8 9 10 ...
##  $ group  : Factor w/ 3 levels "rapamycin+sevoflurane",..: 2 2 2 2 2 2 2 2 2 2 ...
##  $ OCR    : int  86 85 90 75 56 75 97 66 51 83 ...
```

4 Explorative data analysis with graphics

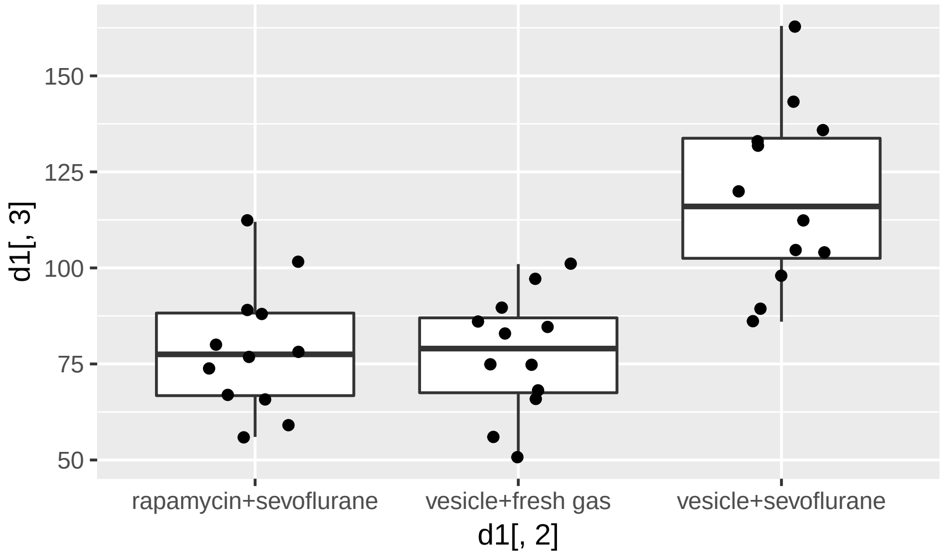

5 Easystat function developed by S. Park (available at https://rpubs.com/goodlebang)

6 Statistical Result

```r
easystat(d1)
```

```
## 1. Normality assumption test by Shapiro_Wilk test is
##  p = 0.887
##  Normality assumption was not rejected
## 2. Equal variance test by Bartlett test is
##  p = 0.337
##  Equal variance assumption was not rejected
## 3. The result of anova is
##  p = 0.0000
##  A statistically significant difference exist between groups
```

```
##   Tukey multiple comparisons of means
##     95% family-wise confidence level
##
## Fit: aov(formula = d1[, 3] ~ d1[, 2], data = d1)
##
## $`d1[, 2]`
##                                              diff       lwr      upr
## vesicle+fresh gas-rapamycin+sevoflurane   -1.25000 -20.17778 17.67778
## vesicle+sevoflurane-rapamycin+sevoflurane 39.41667  20.48888 58.34445
## vesicle+sevoflurane-vesicle+fresh gas     40.66667  21.73888 59.59445
##                                              p adj
## vesicle+fresh gas-rapamycin+sevoflurane   0.9856334
## vesicle+sevoflurane-rapamycin+sevoflurane 0.0000389
## vesicle+sevoflurane-vesicle+fresh gas     0.0000242
```
