# Supplementary material for: The mTOR Inhibitor Rapamycin Prevents General Anesthesia-Induced Changes in Synaptic Transmission and Mitochondrial Respiration in Late Postnatal Mice
Source: Front Cell Neurosci. 2020 Jan 28;14:4. doi: 10.3389/fncel.2020.00004 (PMC6997293; doi:10.3389/fncel.2020.00004)
Supplement: Supplementary file 3 [file Data_Sheet_3.PDF]

Fig3_male PSD95 Data analysis using R

By Sangil Park & Boohwi Hong

1 Package install

```
Packages <- c("tidyverse", "car", "dunn.test","onewaytests","FSA")
lapply(Packages, library, character.only = TRUE)
```

2 Data import

```
d1<- read.csv("/Users/koho0/Desktop/stats/fig3_male PSD95.csv")
```

3 Data structure

```
str(d1)
```

```
## 'data.frame':    14 obs. of  3 variables:
##  $ subject: int  1 2 3 4 5 6 7 8 9 10 ...
##  $ group  : Factor w/ 3 levels "rapamycin+sevoflurane",..: 2 2 2 2 2 3 3 3 3 1 ...
##  $ PSD95  : num  1.53 0.683 0.786 0.882 1.12 ...
```

4 Explorative data analysis with graphics

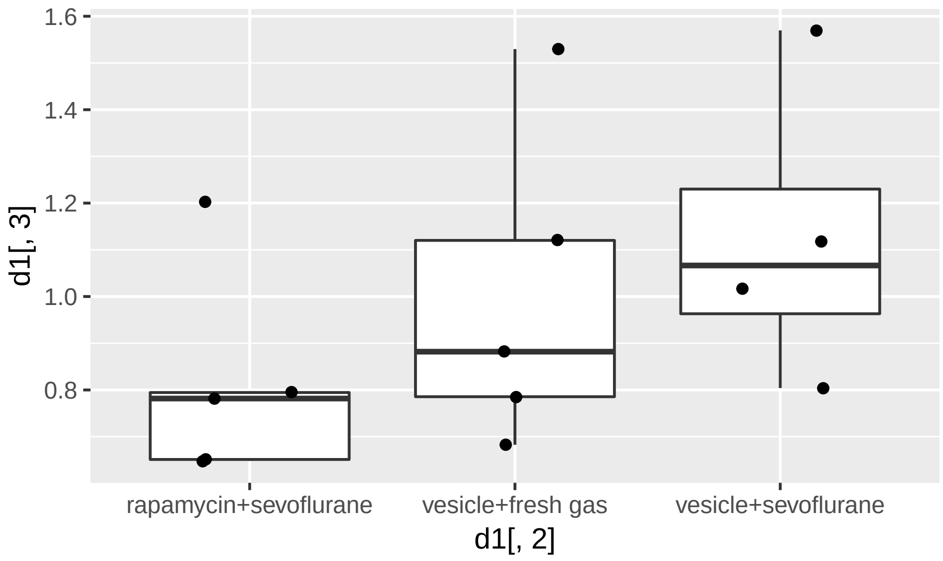

5 Easystat function developed by S. Park (available at https://rpubs.com/goodlebang)

6 Statistical Result

```
easystat(d1)
```

```
## 1. Normality assumption test by Shapiro_Wilk test is
##  p = 0.063
##  Normality assumption was not rejected
## 2. Equal variance test by Bartlett test is
##  p = 0.744
##  Equal variance assumption was not rejected
## 3. The result of anova is
##  p = 0.3245
##  A statistically significant difference do not exist between groups
```

Fig3_male GluA1 Data analysis using R

By Sangil Park & Boohwi Hong

1 Package install

```
Packages <- c("tidyverse", "car", "dunn.test","onewaytests","FSA")
lapply(Packages, library, character.only = TRUE)
```

2 Data import

```
d1<- read.csv("/Users/koho0/Desktop/stats/fig3_male GluA1.csv")
```

3 Data structure

```
str(d1)
```

```
## 'data.frame':    14 obs. of  3 variables:
##  $ subject: int  1 2 3 4 5 6 7 8 9 10 ...
##  $ group  : Factor w/ 3 levels "rapamycin+sevoflurane",..: 2 2 2 2 2 3 3 3 3 1 ...
##  $ GluA1  : num  0.415 0.648 1.129 1.59 1.217 ...
```

4 Explorative data analysis with graphics

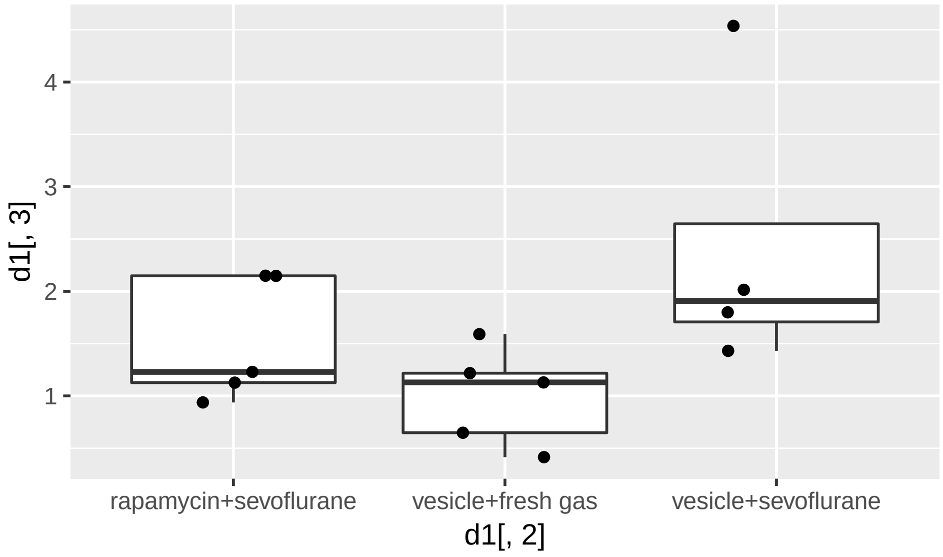

5 Easystat function developed by S. Park (available at https://rpubs.com/goodlebang)

6 Statistical Result

```
easystat(d1)
```

```
## 1. Normality assumption test by Shapiro_Wilk test is
##  p = 0.046
##  Normality assumption was rejected
## 2. The result of Kruskall_Wallis test:
##  p = 0.089
##  A statistically significant difference do not exist between groups
```

Fig3_male GluA2 Data analysis using R

By Sangil Park & Boohwi Hong

1 Package install

```
Packages <- c("tidyverse", "car", "dunn.test","onewaytests","FSA")
lapply(Packages, library, character.only = TRUE)
```

2 Data import

```
d1<- read.csv("/Users/koho0/Desktop/stats/fig3_male GluA2.csv")
```

3 Data structure

```
str(d1)
```

```
## 'data.frame':    14 obs. of  3 variables:
##  $ subject: int  1 2 3 4 5 6 7 8 9 10 ...
##  $ group  : Factor w/ 3 levels "rapamycin+sevoflurane",..: 2 2 2 2 2 3 3 3 3 1 ...
##  $ GluA2  : num  1.019 0.746 0.933 1.18 1.122 ...
```

4 Explorative data analysis with graphics

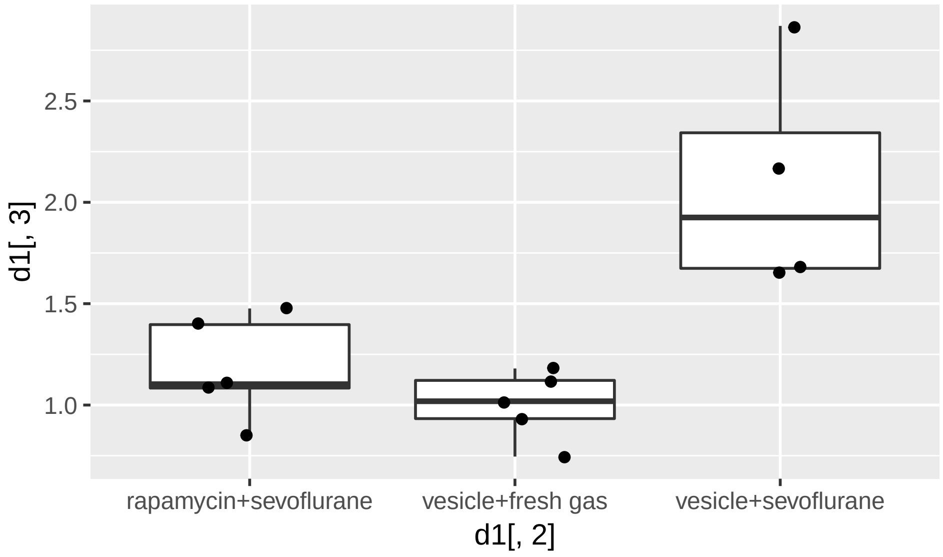

5 Easystat function developed by S. Park (available at https://rpubs.com/goodlebang)

6 Statistical Result

```r
easystat(d1)
```

```
## 1. Normality assumption test by Shapiro_Wilk test is
##  p = 0.451
##  Normality assumption was not rejected
## 2. Equal variance test by Bartlett test is
##  p = 0.091
##  Equal variance assumption was not rejected
## 3. The result of anova is
##  p = 0.0017
##  A statistically significant difference exist between groups
```

```
##   Tukey multiple comparisons of means
##     95% family-wise confidence level
##
## Fit: aov(formula = d1[, 3] ~ d1[, 2], data = d1)
##
## $`d1[, 2]`
##                                                diff        lwr       upr
## vesicle+fresh gas-rapamycin+sevoflurane   -0.1821204 -0.7806618 0.4164209
## vesicle+sevoflurane-rapamycin+sevoflurane  0.9098981  0.2750491 1.5447470
## vesicle+sevoflurane-vesicle+fresh gas      1.0920185  0.4571695 1.7268674
##                                              p adj
## vesicle+fresh gas-rapamycin+sevoflurane   0.6978609
## vesicle+sevoflurane-rapamycin+sevoflurane 0.0067426
## vesicle+sevoflurane-vesicle+fresh gas     0.0018793
```

Fig3_male GAD65 Data analysis using R

By Sangil Park & Boohwi Hong

1 Package install

```
Packages <- c("tidyverse", "car", "dunn.test","onewaytests","FSA")
lapply(Packages, library, character.only = TRUE)
```

2 Data import

```
d1<- read.csv("/Users/koho0/Desktop/stats/fig3_male GAD65.csv")
```

3 Data structure

```
str(d1)
```

```
## 'data.frame':    14 obs. of  3 variables:
##  $ subject: int  1 2 3 4 5 6 7 8 9 10 ...
##  $ group  : Factor w/ 3 levels "rapamycin+sevoflurane",..: 2 2 2 2 2 3 3 3 3 1 ...
##  $ GAD65  : num  1.521 0.749 0.944 0.894 0.892 ...
```

4 Explorative data analysis with graphics

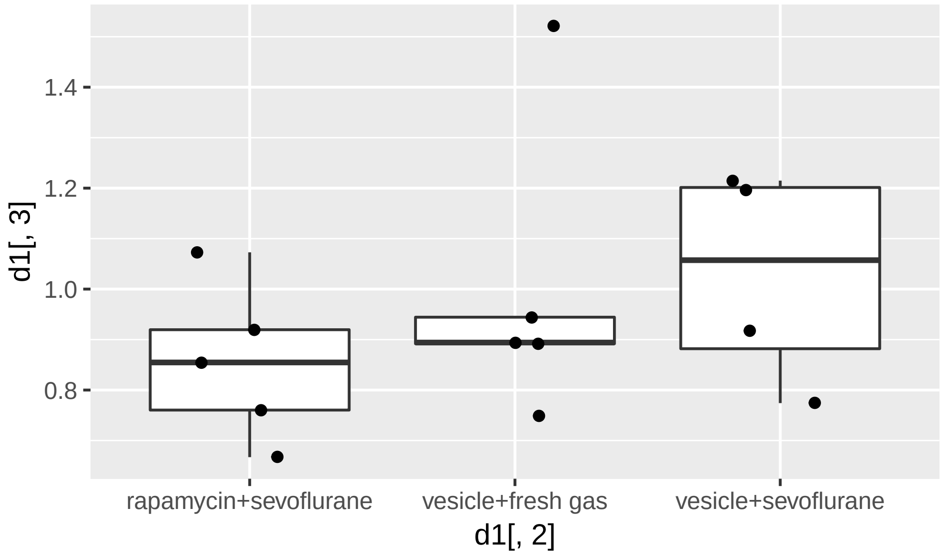

5 Easystat function developed by S. Park (available at https://rpubs.com/goodlebang)

6 Statistical Result

```
easystat(d1)
```

```
## 1. Normality assumption test by Shapiro_Wilk test is
##  p = 0.131
##  Normality assumption was not rejected
## 2. Equal variance test by Bartlett test is
##  p = 0.474
##  Equal variance assumption was not rejected
## 3. The result of anova is
##  p = 0.4988
##  A statistically significant difference do not exist between groups
```

Fig3_female PSD95 Data analysis using R

By Sangil Park & Boohwi Hong

1 Package install

```
Packages <- c("tidyverse", "car", "dunn.test","onewaytests","FSA")
lapply(Packages, library, character.only = TRUE)
```

2 Data import

```
d1<- read.csv("/Users/koho0/Desktop/stats/fig3_female PSD95.csv")
```

3 Data structure

```
str(d1)
```

```
## 'data.frame':    14 obs. of  3 variables:
##  $ subject: int  1 2 3 4 5 6 7 8 9 10 ...
##  $ group  : Factor w/ 3 levels "rapamycin+sevoflurane",..: 2 2 2 2 3 3 3 3 3 1 ...
##  $ PSD95  : num  1.043 0.808 1.012 1.137 0.82 ...
```

4 Explorative data analysis with graphics

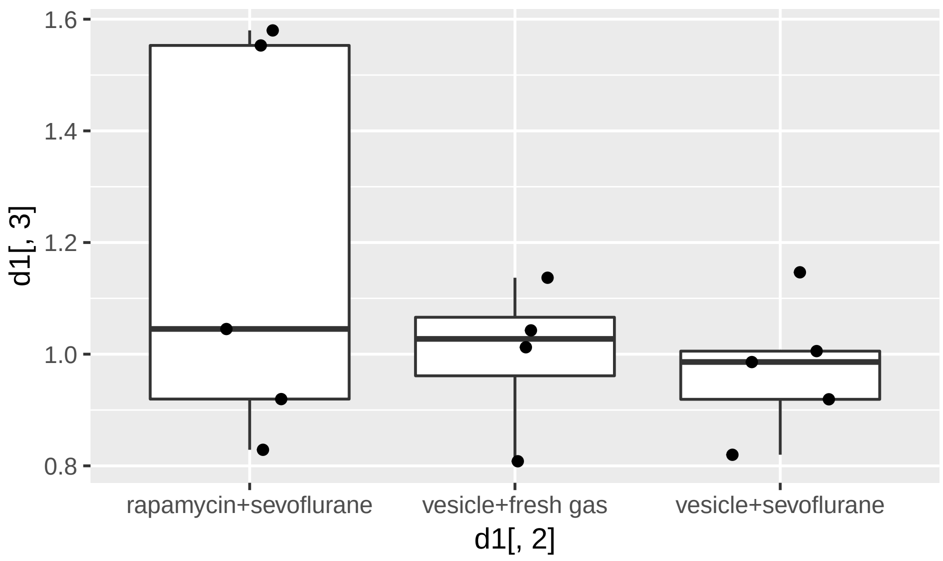

5 Easystat function developed by S. Park (available at https://rpubs.com/goodlebang)

6 Statistical Result

```r
easystat(d1)
```

```
## 1. Normality assumption test by Shapiro_Wilk test is
##  p = 0.8
##  Normality assumption was not rejected
## 2. Equal variance test by Bartlett test is
##  p = 0.086
##  Equal variance assumption was not rejected
## 3. The result of anova is
##  p = 0.3565
##  A statistically significant difference do not exist between groups
```

Fig3_female GluA1 Data analysis using R

By Sangil Park & Boohwi Hong

1 Package install

```
Packages <- c("tidyverse", "car", "dunn.test","onewaytests","FSA")
lapply(Packages, library, character.only = TRUE)
```

2 Data import

```
d1<- read.csv("/Users/koho0/Desktop/stats/fig3_female GluA1.csv")
```

3 Data structure

```
str(d1)
```

```
## 'data.frame':    14 obs. of  3 variables:
##  $ subject: int  1 2 3 4 5 6 7 8 9 10 ...
##  $ group  : Factor w/ 3 levels "rapamycin+sevoflurane",..: 2 2 2 2 3 3 3 3 3 1 ...
##  $ GluA1  : num  0.716 0.743 0.902 1.64 0.696 ...
```

4 Explorative data analysis with graphics

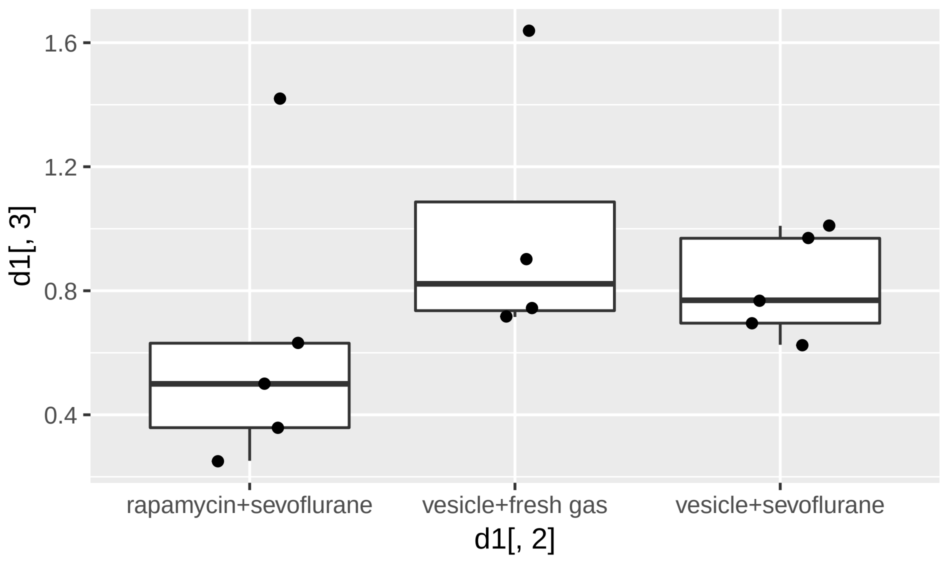

5 Easystat function developed by S. Park (available at https://rpubs.com/goodlebang)

6 Statistical Result

```r
easystat(d1)
```

```
## 1. Normality assumption test by Shapiro_Wilk test is
##  p = 0.019
##  Normality assumption was rejected
## 2. The result of Kruskall_Wallis test:
##  p = 0.176
##  A statistically significant difference do not exist between groups
```

Fig3_female GluA2 Data analysis using R

By Sangil Park & Boohwi Hong

1 Package install

```
Packages <- c("tidyverse", "car", "dunn.test","onewaytests","FSA")
lapply(Packages, library, character.only = TRUE)
```

2 Data import

```
d1<- read.csv("/Users/koho0/Desktop/stats/fig3_female GluA2.csv")
```

3 Data structure

```
str(d1)
```

```
## 'data.frame':    14 obs. of  3 variables:
##  $ subject: int  1 2 3 4 5 6 7 8 9 10 ...
##  $ group  : Factor w/ 3 levels "rapamycin+sevoflurane",..: 2 2 2 2 3 3 3 3 3 1 ...
##  $ GluA2  : num  0.815 0.691 0.964 1.53 0.765 ...
```

4 Explorative data analysis with graphics

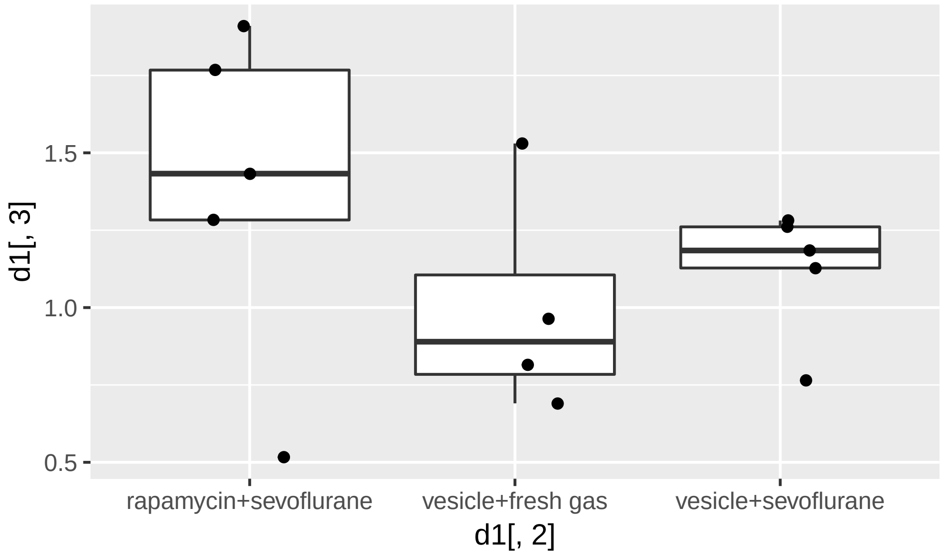

5 Easystat function developed by S. Park (available at https://rpubs.com/goodlebang)

6 Statistical Result

```r
easystat(d1)
```

```
## 1. Normality assumption test by Shapiro_Wilk test is
##  p = 0.51
##  Normality assumption was not rejected
## 2. Equal variance test by Bartlett test is
##  p = 0.236
##  Equal variance assumption was not rejected
## 3. The result of anova is
##  p = 0.3725
##  A statistically significant difference do not exist between groups
```

Fig3_female GAD65 Data analysis using R

By Sangil Park & Boohwi Hong

1 Package install

```
Packages <- c("tidyverse", "car", "dunn.test","onewaytests","FSA")
lapply(Packages, library, character.only = TRUE)
```

2 Data import

```
d1<- read.csv("/Users/koho0/Desktop/stats/fig3_female GAD65.csv")
```

3 Data structure

```
str(d1)
```

```
## 'data.frame':    14 obs. of  3 variables:
##  $ subject: int  1 2 3 4 5 6 7 8 9 10 ...
##  $ group  : Factor w/ 3 levels "rapamycin+sevoflurane",..: 2 2 2 2 3 3 3 3 3 1 ...
##  $ GAD65  : num  0.909 1.009 1.002 1.08 0.673 ...
```

4 Explorative data analysis with graphics

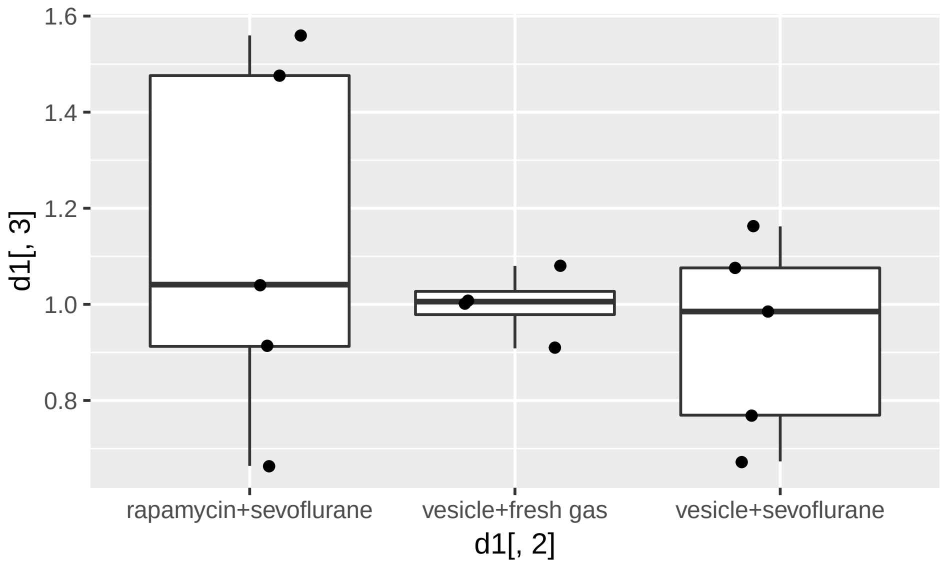

5 Easystat function developed by S. Park (available at https://rpubs.com/goodlebang)

6 Statistical Result

```
easystat(d1)
```

```
## 1. Normality assumption test by Shapiro_Wilk test is
##  p = 0.999
##  Normality assumption was not rejected
## 2. Equal variance test by Bartlett test is
##  p = 0.046
##  Equal variance assumption was rejected
## 3. The result of Welch ANOVA is
##  p = 0.630
##  A statistically significant difference do not exist between groups
```
