# Supplementary material for: The mTOR Inhibitor Rapamycin Prevents General Anesthesia-Induced Changes in Synaptic Transmission and Mitochondrial Respiration in Late Postnatal Mice
Source: Front Cell Neurosci. 2020 Jan 28;14:4. doi: 10.3389/fncel.2020.00004 (PMC6997293; doi:10.3389/fncel.2020.00004)
Supplement: Supplementary file 4 [file Data_Sheet_4.PDF]

Fig4_male mEPSC amplitude Data analysis using R

By Sangil Park & Boohwi Hong

1 Package install

```
Packages <- c("tidyverse", "car", "dunn.test","onewaytests","FSA")
lapply(Packages, library, character.only = TRUE)
```

2 Data import

```
d1<- read.csv("/Users/koho0/Desktop/stats/fig4_male mEPSC amplitude.csv")
```

3 Data structure

```
str(d1)
```

```
## 'data.frame':    55 obs. of  3 variables:
##  $ subject: int  1 2 3 4 5 6 7 8 9 10 ...
##  $ group  : Factor w/ 3 levels "rapamycin+sevoflurane",..: 2 2 2 2 2 2 2 2 2 2 ...
##  $ ampl   : num  21 21.4 30.5 18.4 18.4 ...
```

4 Explorative data analysis with graphics

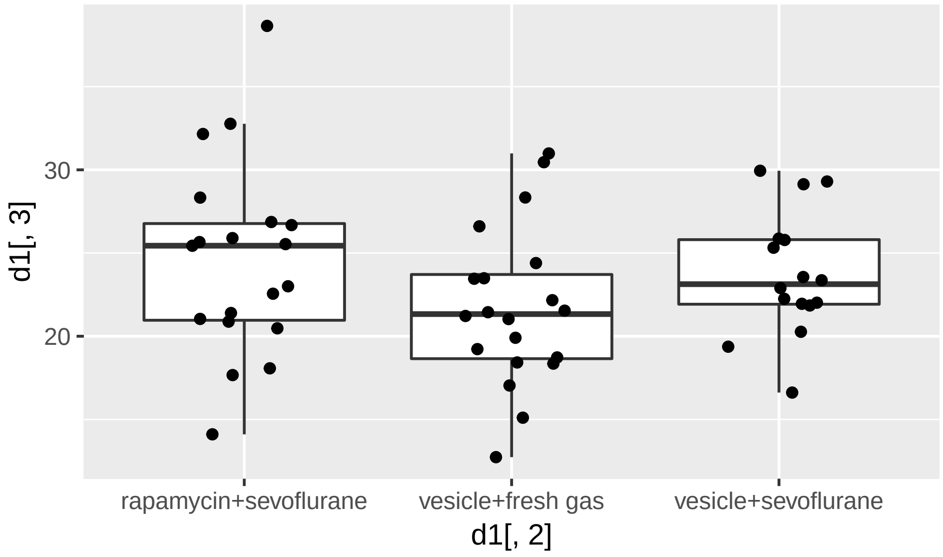

5 Easystat function developed by S. Park (available at https://rpubs.com/goodlebang)

6 Statistical Result

```r
easystat(d1)
```

```
## 1. Normality assumption test by Shapiro_Wilk test is
##  p = 0.495
##  Normality assumption was not rejected
## 2. Equal variance test by Bartlett test is
##  p = 0.206
##  Equal variance assumption was not rejected
## 3. The result of anova is
##  p = 0.1852
##  A statistically significant difference do not exist between groups
```

Fig4_male mEPSC freq Data analysis using R

By Sangil Park & Boohwi Hong

1 Package install

```
Packages <- c("tidyverse", "car", "dunn.test","onewaytests","FSA")
lapply(Packages, library, character.only = TRUE)
```

2 Data import

```
d1<- read.csv("/Users/koho0/Desktop/stats/fig4_male mEPSC freq.csv")
```

3 Data structure

```
str(d1)
```

```
## 'data.frame':    55 obs. of  3 variables:
##  $ subject: int  1 2 3 4 5 6 7 8 9 10 ...
##  $ group  : Factor w/ 3 levels "rapamycin+sevoflurane",..: 2 2 2 2 2 2 2 2 2 2 ...
##  $ freq   : num  0.1 0.0417 0.0583 0.0417 0.05 ...
```

4 Explorative data analysis with graphics

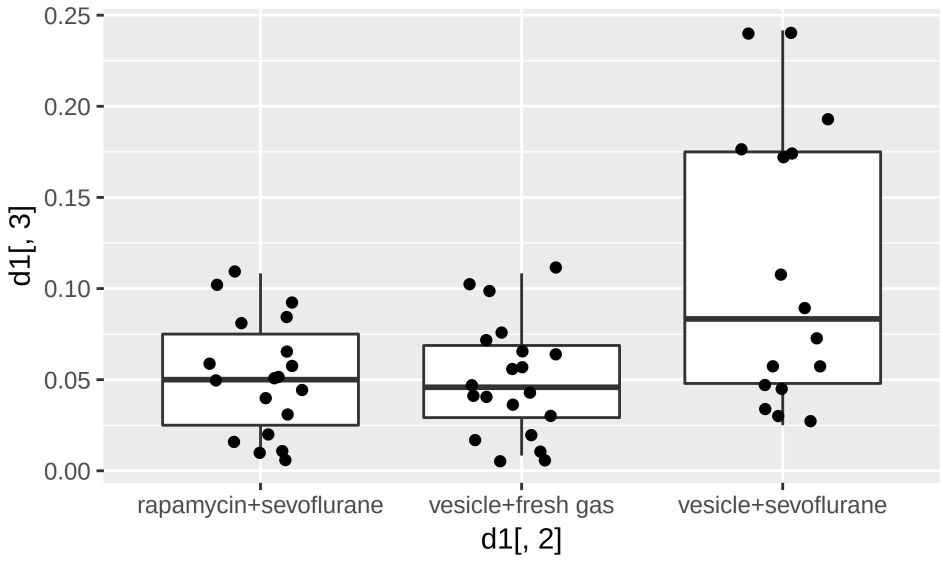

5 Easystat function developed by S. Park (available at https://rpubs.com/goodlebang)

6 Statistical Result

```r
easystat(d1)
```

```
## 1. Normality assumption test by Shapiro_Wilk test is
##  p = 0.107
##  Normality assumption was not rejected
## 2. Equal variance test by Bartlett test is
##  p = 0.000
##  Equal variance assumption was rejected
## 3. The result of Welch ANOVA is
##  p = 0.020
##  A statistically significant difference exist between groups
```

```
##   Tukey multiple comparisons of means
##     95% family-wise confidence level
##
## Fit: aov(formula = d1[, 3] ~ d1[, 2])
##
## $`d1[, 2]`
##                                                     diff         lwr
## vesicle+fresh gas-rapamycin+sevoflurane   -0.0008991228 -0.03876131
## vesicle+sevoflurane-rapamycin+sevoflurane  0.0596217106  0.01952005
## vesicle+sevoflurane-vesicle+fresh gas      0.0605208334  0.02088012
##                                                  upr     p adj
## vesicle+fresh gas-rapamycin+sevoflurane   0.03696306 0.9981920
## vesicle+sevoflurane-rapamycin+sevoflurane 0.09972337 0.0021012
## vesicle+sevoflurane-vesicle+fresh gas     0.10016154 0.0015669
```

Fig4_female mEPSC amplitude Data analysis using R

By Sangil Park & Boohwi Hong

1 Package install

```
Packages <- c("tidyverse", "car", "dunn.test","onewaytests","FSA")
lapply(Packages, library, character.only = TRUE)
```

2 Data import

```
d1<- read.csv("/Users/koho0/Desktop/stats/fig4_female mEPSC amplitude.csv")
```

3 Data structure

```
str(d1)
```

```
## 'data.frame':    57 obs. of  3 variables:
##  $ subject: int  1 2 3 4 5 6 7 8 9 10 ...
##  $ group  : Factor w/ 3 levels "rapamycin+sevoflurane",..: 2 2 2 2 2 2 2 2 2 2 ...
##  $ ampl   : num  17.8 32.6 15.2 17.3 19.5 ...
```

4 Explorative data analysis with graphics

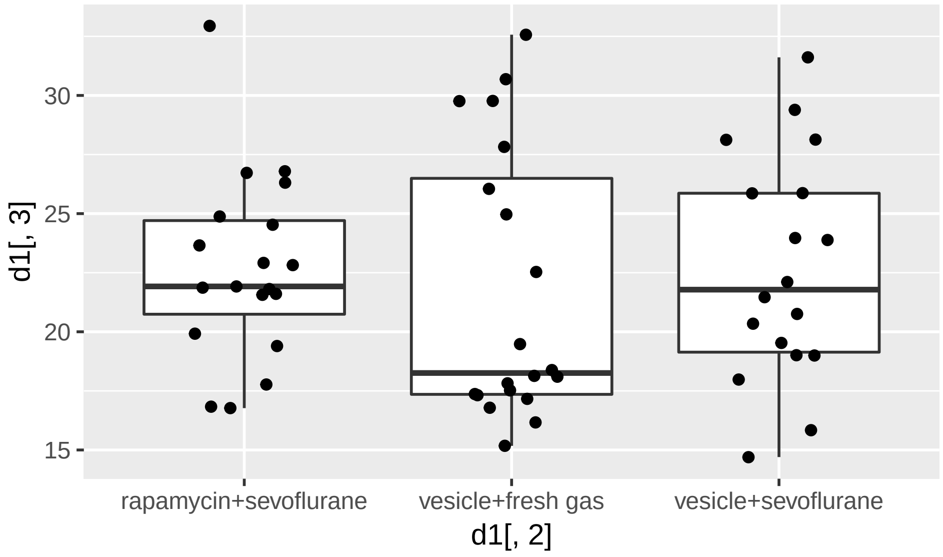

5 Easystat function developed by S. Park (available at https://rpubs.com/goodlebang)

6 Statistical Result

```r
easystat(d1)
```

```
## 1. Normality assumption test by Shapiro_Wilk test is
##  p = 0.018
##  Normality assumption was rejected
## 2. The result of Kruskall_Wallis test:
##  p = 0.531
##  A statistically significant difference do not exist between groups
```

Fig4_female mEPSC freq Data analysis using R

By Sangil Park & Boohwi Hong

1 Package install

```
Packages <- c("tidyverse", "car", "dunn.test","onewaytests","FSA")
lapply(Packages, library, character.only = TRUE)
```

2 Data import

```
d1<- read.csv("/Users/koho0/Desktop/stats/fig4_female mEPSC freq.csv")
```

3 Data structure

```
str(d1)
```

```
## 'data.frame':    57 obs. of  3 variables:
##  $ subject: int  1 2 3 4 5 6 7 8 9 10 ...
##  $ group  : Factor w/ 3 levels "rapamycin+sevoflurane",..: 2 2 2 2 2 2 2 2 2 2 ...
##  $ freq   : num  0.025 0.03333 0.00833 0.01667 0.01667 ...
```

4 Explorative data analysis with graphics

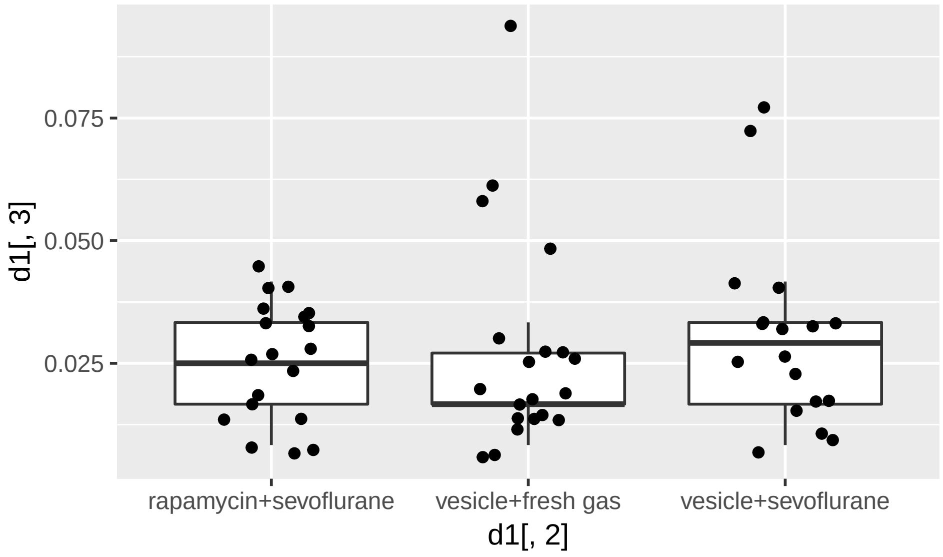

5 Easystat function developed by S. Park (available at https://rpubs.com/goodlebang)

6 Statistical Result

```r
easystat(d1)
```

```
## 1. Normality assumption test by Shapiro_Wilk test is
##  p = 0
##  Normality assumption was rejected
## 2. The result of Kruskall_Wallis test:
##  p = 0.608
##  A statistically significant difference do not exist between groups
```
