# Supplementary material for: The mTOR Inhibitor Rapamycin Prevents General Anesthesia-Induced Changes in Synaptic Transmission and Mitochondrial Respiration in Late Postnatal Mice
Source: Front Cell Neurosci. 2020 Jan 28;14:4. doi: 10.3389/fncel.2020.00004 (PMC6997293; doi:10.3389/fncel.2020.00004)
Supplement: Supplementary file 5 [file Data_Sheet_5.PDF]

Fig5_male mIPSC amplitude Data analysis using R

By Sangil Park & Boohwi Hong

1 Package install

```
Packages <- c("tidyverse", "car", "dunn.test","onewaytests","FSA")
lapply(Packages, library, character.only = TRUE)
```

2 Data import

```
d1<- read.csv("/Users/koho0/Desktop/stats/fig5_male mIPSC amplitude.csv")
```

3 Data structure

```
str(d1)
```

```
## 'data.frame':    64 obs. of  3 variables:
##  $ subject: int  1 2 3 4 5 6 7 8 9 10 ...
##  $ group  : Factor w/ 3 levels "rapamycin+sevoflurane",..: 2 2 2 2 2 2 2 2 2 2 ...
##  $ ampl   : num  35.9 48.3 49.1 55.9 39.4 ...
```

4 Explorative data analysis with graphics

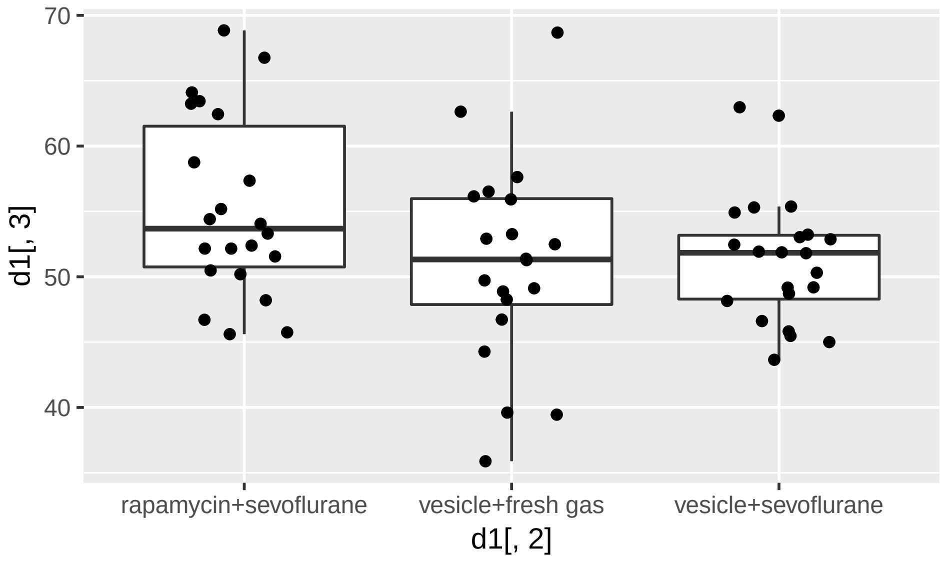

5 Easystat function developed by S. Park (available at https://rpubs.com/goodlebang)

6 Statistical Result

```
easystat(d1)
```

```
## 1. Normality assumption test by Shapiro_Wilk test is
##  p = 0.766
##  Normality assumption was not rejected
## 2. Equal variance test by Bartlett test is
##  p = 0.143
##  Equal variance assumption was not rejected
## 3. The result of anova is
##  p = 0.0692
##  A statistically significant difference do not exist between groups
```

Fig5_male mIPSC freq Data analysis using R

By Sangil Park & Boohwi Hong

1 Package install

```
Packages <- c("tidyverse", "car", "dunn.test","onewaytests","FSA")
lapply(Packages, library, character.only = TRUE)
```

2 Data import

```
d1<- read.csv("/Users/koho0/Desktop/stats/fig5_male mIPSC freq.csv")
```

3 Data structure

```
str(d1)
```

```
## 'data.frame':    64 obs. of  3 variables:
##  $ subject: int  1 2 3 4 5 6 7 8 9 10 ...
##  $ group  : Factor w/ 3 levels "rapamycin+sevoflurane",..: 2 2 2 2 2 2 2 2 2 2 ...
##  $ freq   : num  3.45 5.28 6.99 5.47 5.95 ...
```

4 Explorative data analysis with graphics

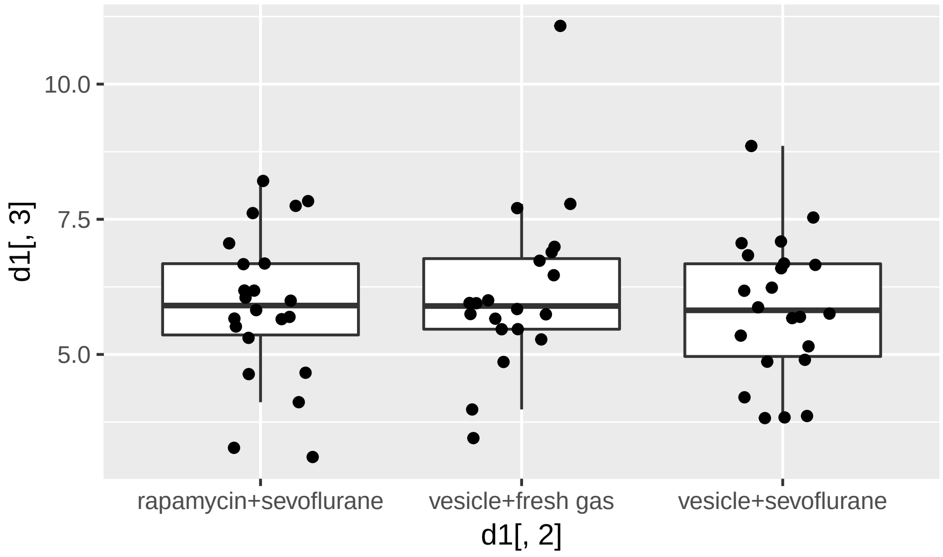

5 Easystat function developed by S. Park (available at https://rpubs.com/goodlebang)

6 Statistical Result

```
easystat(d1)
```

```
## 1. Normality assumption test by Shapiro_Wilk test is
##  p = 0.104
##  Normality assumption was not rejected
## 2. Equal variance test by Bartlett test is
##  p = 0.681
##  Equal variance assumption was not rejected
## 3. The result of anova is
##  p = 0.7616
##  A statistically significant difference do not exist between groups
```

Fig5_female mIPSC amplitude Data analysis using R

By Sangil Park & Boohwi Hong

1 Package install

```
Packages <- c("tidyverse", "car", "dunn.test","onewaytests","FSA")
lapply(Packages, library, character.only = TRUE)
```

2 Data import

```
d1<- read.csv("/Users/koho0/Desktop/stats/fig5_female mIPSC amplitude.csv")
```

3 Data structure

```
str(d1)
```

```
## 'data.frame':    65 obs. of  3 variables:
##  $ subject: int  1 2 3 4 5 6 7 8 9 10 ...
##  $ group  : Factor w/ 3 levels "rapamycin+sevoflurane",..: 2 2 2 2 2 2 2 2 2 2 ...
##  $ ampl   : num  46.1 36.4 30.5 31.1 54.9 ...
```

4 Explorative data analysis with graphics

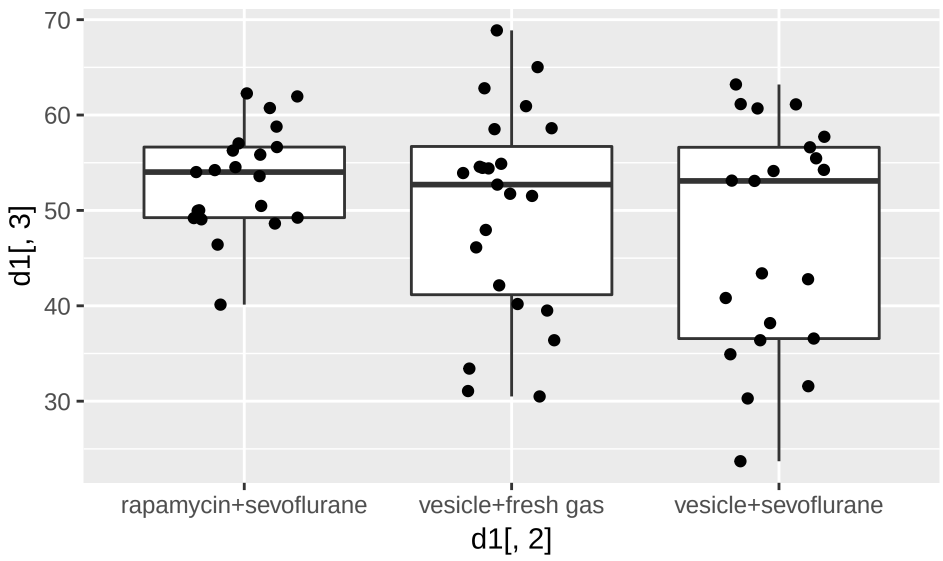

5 Easystat function developed by S. Park (available at https://rpubs.com/goodlebang)

6 Statistical Result

```
easystat(d1)
```

```
## 1. Normality assumption test by Shapiro_Wilk test is
##  p = 0.322
##  Normality assumption was not rejected
## 2. Equal variance test by Bartlett test is
##  p = 0.003
##  Equal variance assumption was rejected
## 3. The result of Welch ANOVA is
##  p = 0.085
##  A statistically significant difference do not exist between groups
```

Fig5_female mIPSC freq Data analysis using R

By Sangil Park & Boohwi Hong

1 Package install

```
Packages <- c("tidyverse", "car", "dunn.test","onewaytests","FSA")
lapply(Packages, library, character.only = TRUE)
```

2 Data import

```
d1<- read.csv("/Users/koho0/Desktop/stats/fig5_female mIPSC freq.csv")
```

3 Data structure

```
str(d1)
```

```
## 'data.frame':    65 obs. of  3 variables:
##  $ subject: int  1 2 3 4 5 6 7 8 9 10 ...
##  $ group  : Factor w/ 3 levels "rapamycin+sevoflurane",..: 2 2 2 2 2 2 2 2 2 2 ...
##  $ freq   : num  8.68 7.57 7.92 7.25 8.56 ...
```

4 Explorative data analysis with graphics

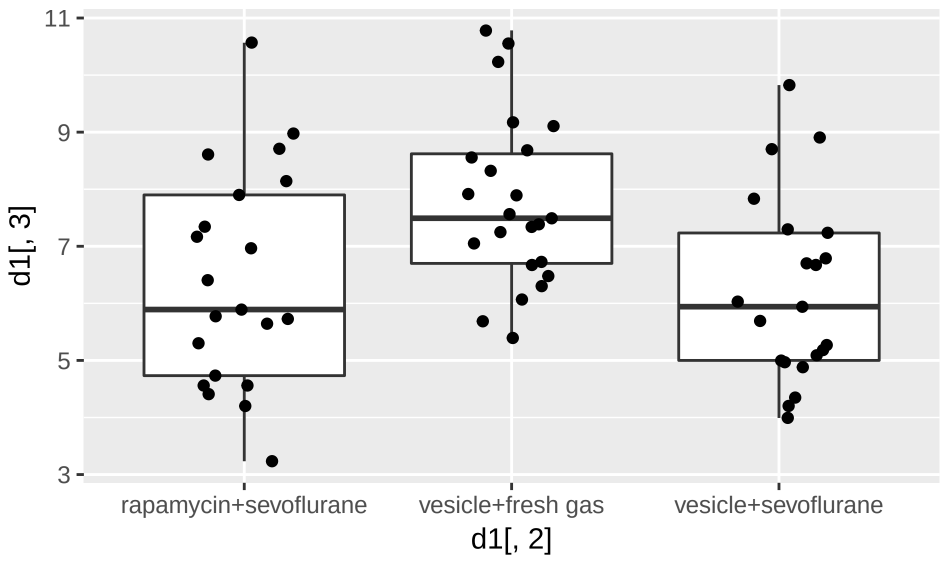

5 Easystat function developed by S. Park (available at https://rpubs.com/goodlebang)

6 Statistical Result

```
easystat(d1)
```

```
## 1. Normality assumption test by Shapiro_Wilk test is
##  p = 0.094
##  Normality assumption was not rejected
## 2. Equal variance test by Bartlett test is
##  p = 0.523
##  Equal variance assumption was not rejected
## 3. The result of anova is
##  p = 0.0057
##  A statistically significant difference exist between groups
```

```
##   Tukey multiple comparisons of means
##     95% family-wise confidence level
##
## Fit: aov(formula = d1[, 3] ~ d1[, 2], data = d1)
##
## $`d1[, 2]`
##                                                 diff       lwr        upr
## vesicle+fresh gas-rapamycin+sevoflurane    1.3468599  0.131854  2.5618658
## vesicle+sevoflurane-rapamycin+sevoflurane -0.2019841 -1.444297  1.0403287
## vesicle+sevoflurane-vesicle+fresh gas     -1.5488440 -2.763850 -0.3338382
##                                               p adj
## vesicle+fresh gas-rapamycin+sevoflurane   0.0263698
## vesicle+sevoflurane-rapamycin+sevoflurane 0.9195290
## vesicle+sevoflurane-vesicle+fresh gas     0.0090163
```
