# Supplementary material for: The mTOR Inhibitor Rapamycin Prevents General Anesthesia-Induced Changes in Synaptic Transmission and Mitochondrial Respiration in Late Postnatal Mice
Source: Front Cell Neurosci. 2020 Jan 28;14:4. doi: 10.3389/fncel.2020.00004 (PMC6997293; doi:10.3389/fncel.2020.00004)
Supplement: Supplementary file 6 [file Data_Sheet_6.PDF]

Suppl fig1_male mTor ratio Data analysis using R

By Sangil Park & Boohwi Hong

1 Package install

```
Packages <- c("tidyverse", "car", "dunn.test","onewaytests","FSA")
lapply(Packages, library, character.only = TRUE)
```

2 Data import

```
d1<- read.csv("/Users/koho0/Desktop/stats/suppl fig1_male mTor ratio.csv")
```

3 Data structure

```
str(d1)
```

```
## 'data.frame':    14 obs. of  3 variables:
##  $ subject: int  1 2 3 4 5 6 7 8 9 10 ...
##  $ group  : Factor w/ 3 levels "rapamycin+sevoflurane",..: 2 2 2 2 3 3 3 3 3 1 ...
##  $ ratio  : num  0.609 0.494 1.119 1.777 3.806 ...
```

4 Explorative data analysis with graphics

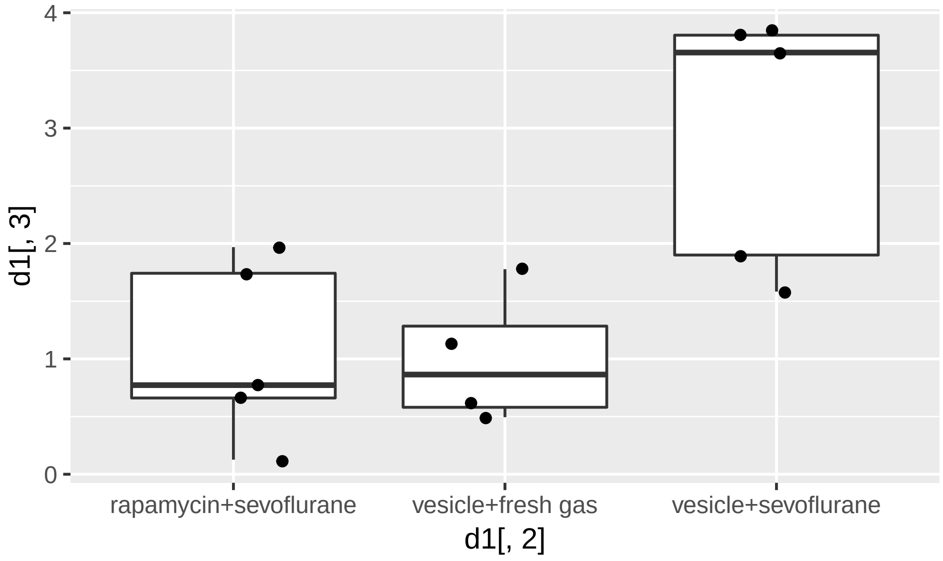

5 Easystat function developed by S. Park (available at https://rpubs.com/goodlebang)

6 Statistical Result

```r
easystat(d1)
```

```
## 1. Normality assumption test by Shapiro_Wilk test is
##  p = 0.079
##  Normality assumption was not rejected
## 2. Equal variance test by Bartlett test is
##  p = 0.529
##  Equal variance assumption was not rejected
## 3. The result of anova is
##  p = 0.0078
##  A statistically significant difference exist between groups
```

```
##   Tukey multiple comparisons of means
##     95% family-wise confidence level
##
## Fit: aov(formula = d1[, 3] ~ d1[, 2], data = d1)
##
## $`d1[, 2]`
##                                                diff        lwr      upr
## vesicle+fresh gas-rapamycin+sevoflurane   -0.0540884 -1.6396911 1.531514
## vesicle+sevoflurane-rapamycin+sevoflurane  1.9029811  0.4080605 3.397902
## vesicle+sevoflurane-vesicle+fresh gas      1.9570695  0.3714668 3.542672
##                                               p adj
## vesicle+fresh gas-rapamycin+sevoflurane   0.9953331
## vesicle+sevoflurane-rapamycin+sevoflurane 0.0140983
## vesicle+sevoflurane-vesicle+fresh gas     0.0168709
```

Suppl fig1_female mTor ratio Data analysis using R

By Sangil Park & Boohwi Hong

1 Package install

```
Packages <- c("tidyverse", "car", "dunn.test","onewaytests","FSA")
lapply(Packages, library, character.only = TRUE)
```

2 Data import

```
d1<- read.csv("/Users/koho0/Desktop/stats/suppl fig1_female mTor ratio.csv")
```

3 Data structure

```
str(d1)
```

```
## 'data.frame':    14 obs. of  3 variables:
##  $ subject: int  1 2 3 4 5 6 7 8 9 10 ...
##  $ group  : Factor w/ 3 levels "rapamycin+sevoflurane",..: 2 2 2 2 3 3 3 3 3 1 ...
##  $ ratio  : num  0.214 0.612 1.545 1.629 2.781 ...
```

4 Explorative data analysis with graphics

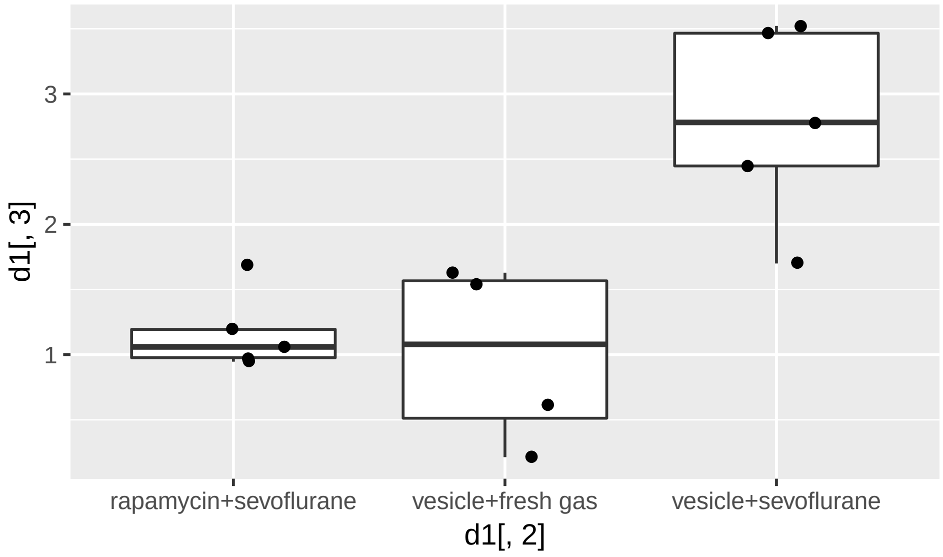

5 Easystat function developed by S. Park (available at https://rpubs.com/goodlebang)

6 Statistical Result

```r
easystat(d1)
```

```
## 1. Normality assumption test by Shapiro_Wilk test is
##  p = 0.351
##  Normality assumption was not rejected
## 2. Equal variance test by Bartlett test is
##  p = 0.244
##  Equal variance assumption was not rejected
## 3. The result of anova is
##  p = 0.0016
##  A statistically significant difference exist between groups
```

```
##   Tukey multiple comparisons of means
##     95% family-wise confidence level
##
## Fit: aov(formula = d1[, 3] ~ d1[, 2], data = d1)
##
## $`d1[, 2]`
##                                               diff        lwr       upr
## vesicle+fresh gas-rapamycin+sevoflurane   -0.171920 -1.2811297 0.9372897
## vesicle+sevoflurane-rapamycin+sevoflurane  1.611072  0.5652996 2.6568454
## vesicle+sevoflurane-vesicle+fresh gas      1.782993  0.6737828 2.8922022
##                                               p adj
## vesicle+fresh gas-rapamycin+sevoflurane   0.9087053
## vesicle+sevoflurane-rapamycin+sevoflurane 0.0041497
## vesicle+sevoflurane-vesicle+fresh gas     0.0030796
```

Suppl fig1_male mEPSC amplitude Data analysis using R

By Sangil Park & Boohwi Hong

1 Package install

```r
Packages <- c("tidyverse", "car", "dunn.test","onewaytests","FSA")
lapply(Packages, library, character.only = TRUE)
```

2 Data import

```r
d1<- read.csv("/Users/koho0/Desktop/stats/suppl fig1_male mEPSC amplitude.csv")
```

3 Data structure

```r
str(d1)
```

```
## 'data.frame':    54 obs. of  3 variables:
##  $ subject: int  1 2 3 4 5 6 7 8 9 10 ...
##  $ group  : Factor w/ 3 levels "rapamycin+sevoflurane",..: 2 2 2 2 2 2 2 2 2 2 ...
##  $ ampl   : num  23.8 29.9 37.2 27 35.8 ...
```

4 Explorative data analysis with graphics

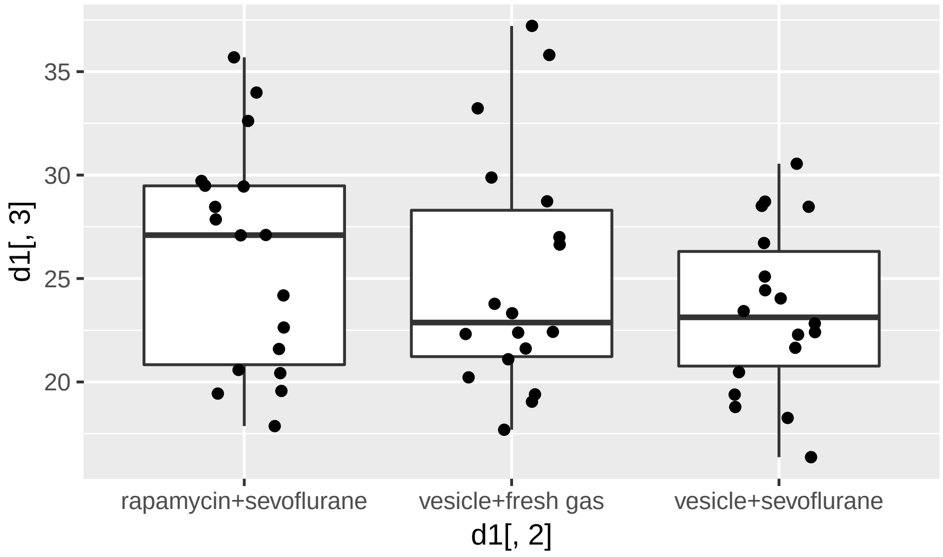

5 Easystat function developed by S. Park (available at https://rpubs.com/goodlebang)

6 Statistical Result

```r
easystat(d1)
```

```
## 1. Normality assumption test by Shapiro_Wilk test is
##  p = 0.126
##  Normality assumption was not rejected
## 2. Equal variance test by Bartlett test is
##  p = 0.309
##  Equal variance assumption was not rejected
## 3. The result of anova is
##  p = 0.3351
##  A statistically significant difference do not exist between groups
```

Suppl fig1_male mEPSC freq Data analysis using R

By Sangil Park & Boohwi Hong

1 Package install

```
Packages <- c("tidyverse", "car", "dunn.test","onewaytests","FSA")
lapply(Packages, library, character.only = TRUE)
```

2 Data import

```
d1<- read.csv("/Users/koho0/Desktop/stats/suppl fig1_male mEPSC freq.csv")
```

3 Data structure

```
str(d1)
```

```
## 'data.frame':    54 obs. of  3 variables:
##  $ subject: int  1 2 3 4 5 6 7 8 9 10 ...
##  $ group  : Factor w/ 3 levels "rapamycin+sevoflurane",..: 2 2 2 2 2 2 2 2 2 2 ...
##  $ freq   : num  0.125 0.2 0.125 0.1167 0.0583 ...
```

4 Explorative data analysis with graphics

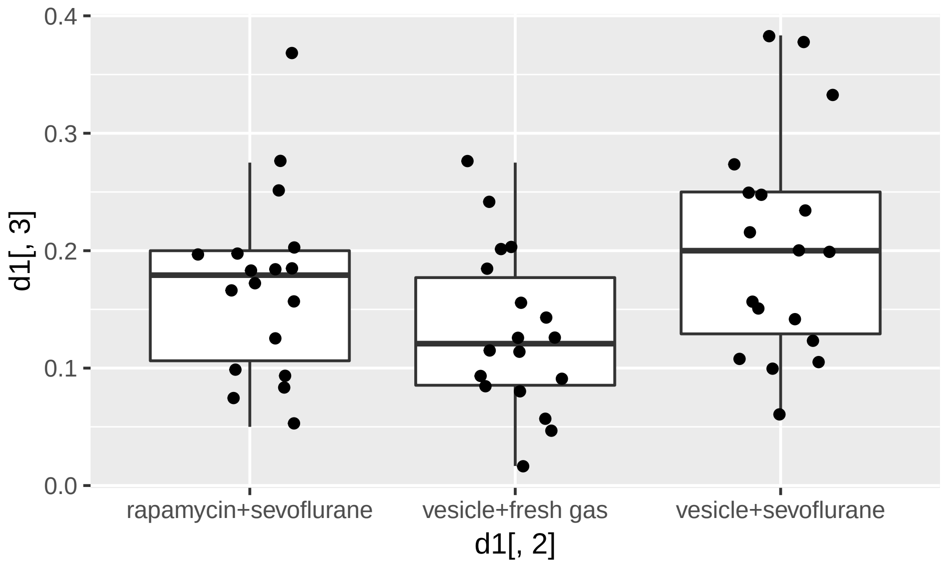

5 Easystat function developed by S. Park (available at https://rpubs.com/goodlebang)

6 Statistical Result

```r
easystat(d1)
```

```
## 1. Normality assumption test by Shapiro_Wilk test is
##  p = 0.144
##  Normality assumption was not rejected
## 2. Equal variance test by Bartlett test is
##  p = 0.391
##  Equal variance assumption was not rejected
## 3. The result of anova is
##  p = 0.0342
##  A statistically significant difference exist between groups
```

```
##   Tukey multiple comparisons of means
##     95% family-wise confidence level
##
## Fit: aov(formula = d1[, 3] ~ d1[, 2], data = d1)
##
## $`d1[, 2]`
##                                                   diff          lwr
## vesicle+fresh gas-rapamycin+sevoflurane   -0.03935185 -0.104719084
## vesicle+sevoflurane-rapamycin+sevoflurane  0.03333333 -0.032033899
## vesicle+sevoflurane-vesicle+fresh gas      0.07268519  0.007317952
##                                                  upr     p adj
## vesicle+fresh gas-rapamycin+sevoflurane   0.02601538 0.3218222
## vesicle+sevoflurane-rapamycin+sevoflurane 0.09870057 0.4406560
## vesicle+sevoflurane-vesicle+fresh gas     0.13805242 0.0260266
```
