# Supplementary figures and images for: The mTOR Inhibitor Rapamycin Prevents General Anesthesia-Induced Changes in Synaptic Transmission and Mitochondrial Respiration in Late Postnatal Mice
Source: Front Cell Neurosci. 2020 Jan 28;14:4. doi: 10.3389/fncel.2020.00004 (PMC6997293; doi:10.3389/fncel.2020.00004)

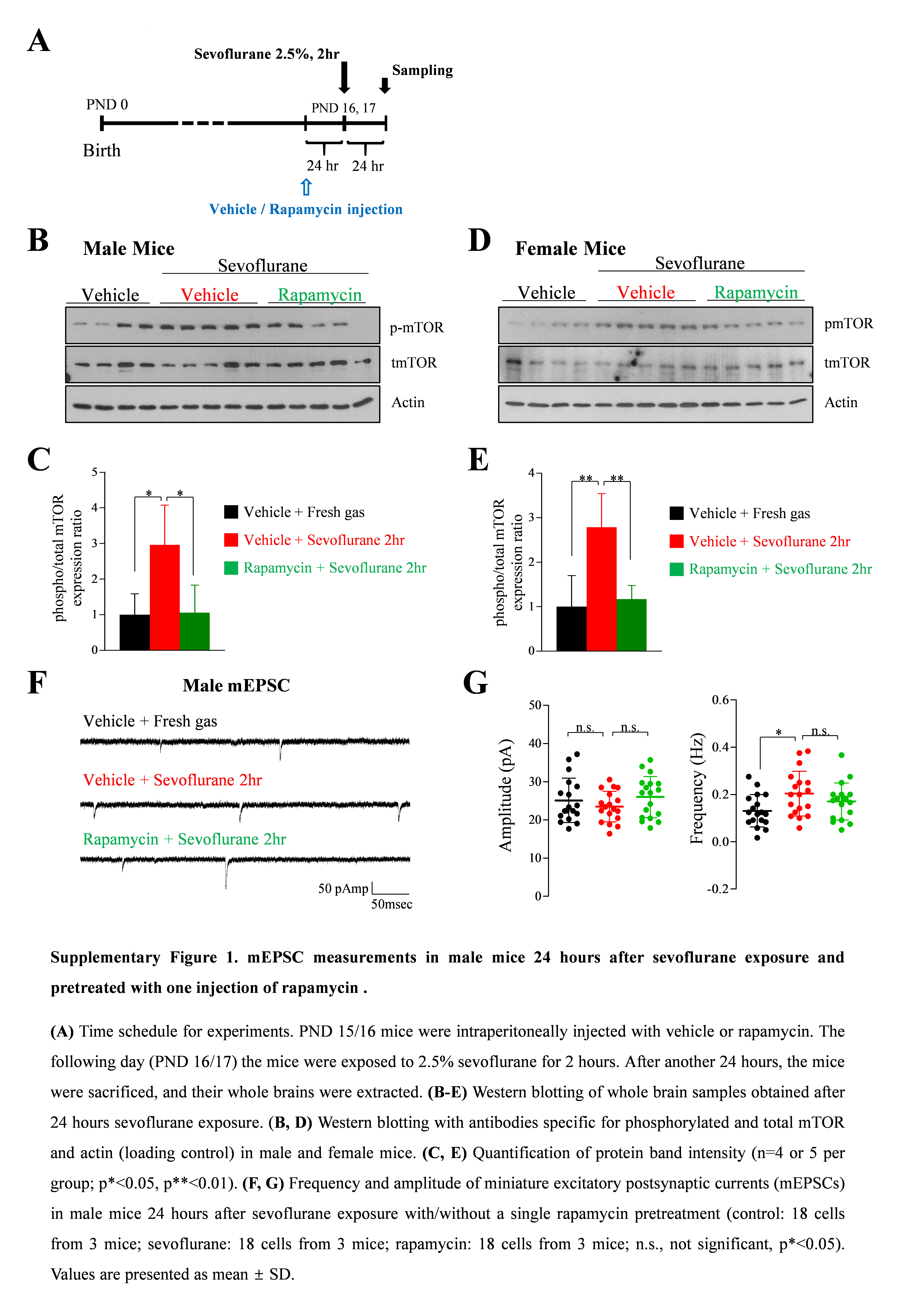

Supplement: Supplementary file 7 [file Image_1.tif]
